# The three-dimensional structure of a proton-pumping pathway, the H-pathway, is evolutionarily conserved in all three families of cytochrome *c* oxidase

**DOI:** 10.3389/fchem.2025.1645343

**Published:** 2026-01-30

**Authors:** Atsuhiro Shimada, Tomitake Tsukihara, Shinya Yoshikawa

**Affiliations:** 1 Department of Applied Life Science, Faculty of Applied Biological Sciences, Gifu University, Gifu, Japan; 2 Department of Life Science, Graduate School of Science, University of Hyogo, Hyogo, Japan; 3 Institute for Protein Research, Osaka University, Osaka, Japan

**Keywords:** bioenergetics, conservativity of H-pathway, cytochrome c oxidase, heme-copper oxidase, proton-pump mechanism, X-ray crystal structure

## Abstract

The O_2_ reduction site of bovine cytochrome *c* oxidase (CcO) comprises two redox-active metal centers: Fe_*a*3_ and Cu_B_. O_2_ is reduced at Fe_*a*3_ by four electrons transferred from cytochrome *c* in the P-side phase. Three proton-conducting pathways, D, K, and H, have been identified. Two distinct proton-pumping mechanisms, the D- and H-pathway mechanisms, proposed over 30 years ago, remain a subject of active debate. The former proposes that D-pathway transfers both pumping and water forming protons, whereas the latter proposes that the H-pathway transfers the pumping protons. CcOs are distributed across all aerobic organisms and are classified into evolutionarily related families: A, B, and C. In this study, we analyzed the common three-dimensional (3D) structural features of representative CcOs from each family to identify the proton-pumping system, assuming that the 3D structures responsible for the fundamental function of CcO, O_2_ reduction coupled with proton pumping, are evolutionarily conserved. Our analysis reveals that the 3D structural elements essential for proton pumping via the H-pathway mechanism are conserved across all three CcO families. These conserved elements include: 1) the site for loading and active release of pumping-protons to the P-side phase; 2) a water channel with a gate which opens to collect pumping protons from the N-side before the catalytic cycle starts and closes during the catalytic cycle to prevent leakage of pumping protons; 3) a water cluster located above the water-channel gate for storing pumping protons delivered from the water channel and transferring them in a timely manner to the proton-loading/release site when the gate is closed; and 4) a pumping-proton pool system, located below the water-channel gate, for the facile supply of protons to the water cluster when the gate opens. This structural conservation suggests that the H-pathway is responsible for proton pumping. Experimental results, supporting the D-pathway mechanism, do not disprove (are consistent with) the H-pathway mechanism. However, structural elements required to prevent pumping-proton leakage to the O_2_-reduction site and to the N-side surface, indispensable for the D-pathway as a proton pumping system, have not been identified experimentally.

## 1 Introduction

Cytochrome *c* oxidase (CcO) is the terminal oxidase of cellular respiration, responsible for reducing molecular oxygen (O_2_) to water, coupled with the generation of a proton-motive force that drives ATP synthase. Among the four redox-active metal sites, hemes *a* and *a*_3_, Cu_A_, and Cu_B_, the O_2_ reduction site, consisting of heme *a*_3_ and Cu_B_, receives electrons from cytochrome *c* in the P-side phase via Cu_A_ and heme *a*. The catalytic cycle of CcO involves six intermediates: R (Fe_*a*3_^2+^, Cu_B_^1+^, Y244OH), A (Fe_*a*3_^2+^–O_2_, Cu_B_^1+^, Y244OH), P_M_((Fe_*a*3_^4+^ = O^2-^, Cu_B_^2+^–OH^-^, Y244O•), F(Fe_*a*3_^4+^ = O^2-^, Cu_B_^2+^–OH^-^, Y244OH), O(Fe_*a*3_^3+^–OH^-^, Cu_B_^2+^–OH^-^, Y244OH), and E (Fe_*a*3_^3+^–OH^-^, Cu_B_^1+^–H_2_O, Y244OH), where Y244O• denotes the neutral radical form of Y244. Each of the four reaction steps from Pm to R (P_M_ → F, F → O, O → E, and E → R) is driven by one-electron donation from cytochrome *c* in the P-side phase, coupled with uptake of two protons from the N-side, for proton pumping and water formation (designated as “pumping proton” and “water-forming proton”, respectively in this article) and with release of one pumping proton to the P-side. The stoichiometry (pumping proton/water-forming proton/electron) in each reaction step has been well established by using the two time-resolved approaches of oxidation of fully reduced CcO with O_2_ (Bloch et al., 2004, Verkhovsky, et al., 1999) and single-electron injection into fixed states of CcO (Siletsky et al., 1999; Siletsky et al., 2004). The protons for formation of water molecules from oxides (O^2-^), generated by complete reduction of O_2_, are transferred from the N-side through two proton-conducting pathways, the D- and K-pathways (Fetter, et al., 1995; Hosler et al., 1996; Konstantinov et al., 1997; Adelroth, et al., 1997; Adelroth, et al., 1998). When fully reduced CcO (Fe_*a*_^2+^, Cu_A_^1+^, Fe_*a*3_^2+^, Cu_B_^1+^, Y244OH) is oxidized with an excess amount of O_2_, P_M_ (Fe_*a*3_^4+^ = O^2-^, Cu_B_^2+^–OH^-^, Y244O•) is rapidly reduced by Fe_*a*_^2+^ to form Pr (Fe_*a*3_^4+^ = O^2-^, Cu_B_^2+^–OH^-^, Y244O^−^). Pr formation is too fast for P_M_ to be detectable. It has been proposed that the water forming proton for the Pr → F transition is transferred to Cu_B_^2+^–OH^-^ rather than to Y244O^−^, since Y244, though located near heme *a*_3_ plane, is unlikely to induce the significantly large absorption spectral change (i.e., the peak shift from 607 nm to 580 nm) (Wikström et al., 2018). However, high resolution X-ray-structural analyses for P_M_ and F forms of bovine CcO show that both forms exhibit an identical structure of the O_2_ reduction site, giving a low barrier hydrogen bond between Fe_*a*3_^4+^ = O^2-^ and Cu_B_^2+^–OH^-^. Thus. Protonation of Cu_B_^2+^–OH^-^ in Pr would induce significant structural changes that disrupts the low barrier hydrogen bond, which would be clearly detectable at the resolution of the X-ray structural analysis performed (Shimada et al., 2017). This structural finding strongly suggests that the O_2_-reduction site is not the site that accepts the water-forming protons for the Pr → F transition. In P_M,_ a clear magnetic coupling is detectable between the Y244-O• and the OH group of the hydroxyfarnesylethyl side chain of the heme. Furthermore, Y244-O• is also magnetically coupled with Fe_*a*3_^4+^ = O^2-^ via a histidine imidazole group and Cu_B_-OH^-^ (Jose, et al., 2021). These structural features strongly suggest that the change in the protonation state of Y244 induces the absorption spectral change in the high-spin heme near Y244. Therefore, the F is written as F (Fe_*a*3_^4+^ = O^2-^, Cu_B_^2+^–OH^-^, Y244OH). A third pathway, the H-pathway, spans CcO from the N- to the P-side and consists of a tandem arrangement of a hydrogen-bond network and a water channel. Two proton pumping mechanisms, designated as the D-pathway and H-pathway mechanisms, were proposed nearly 30 years ago based on mutational and X-ray structural analyses, respectively, and remain subjects of active debate (Adelroth, et al., 1997, Adelroth et al., 1998; Konstantinov et al., 1997; Yoshikawa et al., 1998).

**FIGURE 1:**
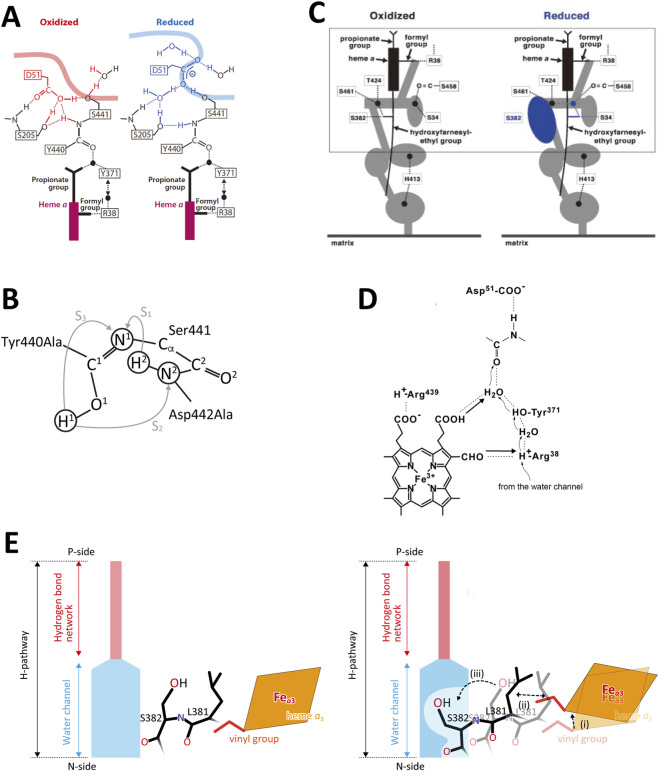
A schematic representation of the structures of bovine CcO critical for the H-pathway mechanism. **(A)** The redox-coupled conformational changes in D51 of the hydrogen-bond network of the H-pathway. The thick curves denote the molecular surface of CcO facing the P-side phase. The filled circles denote the positions of the fixed water molecules. Reprinted from (Shimada et al., 2023a). **(B)** Comparison of the proton transfer pathway through the peptide bond between Y440 and S441, predicted by X-ray structural analysis and marked by an arrow, S_3_, with the multistep proton transfer proposed on the basis of quantitative theoretical analyses, marked by two arrows, S_1_ and S_2_. Reprinted with permission from (Kamiya et al., 2007). **(C)** The redox-coupled 3D structural change in the water channel. The circles and ovals denote cavities in which at least one water molecule can be stored. The thick sticks in the figure denote a side view of the heme *a* plane. R38 denotes the residue at the bottom end of the hydrogen-bond network of the H-pathway as illustrated in Figure 1A. One of the cavities in the reduced state (the blue oval) is eliminated upon oxidation. Reprinted from (Shimada, et al., 2023a). **(D)** Interactions between the hydrogen-bond network of the H-pathway and heme *a*. R38 is at the bottom end of the hydrogen-bond network. Thin arrows denote the direction of transfer of pumping protons from the water channel. A thick arrow denotes possible electrostatic repulsion against proton transfer through the hydrogen-bond network of the H-pathway. Reprinted from (Shimada et al., 2023a). **(E)** The water channel closure mechanism. (left) In the reduced state of heme *a*_3_, the water channel is in the open state and the heme *a*_3_ vinyl group is in van der Waals contact with L381. (right) Upon oxidation of heme *a*_3_, a translational shift of heme *a*_3_ is induced, triggering structural changes in L381 and S382 to close the channel by eliminating a water cavity, as marked by the two dotted arrows. The water cavity is shown in panel C as a blue oval. Reprinted with permission from (Shimada et al., 2017).

The D-pathway mechanism suggests that pumping protons are first transferred from the N-side through the D-pathway to a proton-loading site near the O_2_ reduction site. When water-forming protons are also transferred through the D-pathway, electrostatic repulsion between the two proton types triggers the release of the pumping protons to the P-side phase (Wikström et al., 2018). While this model is widely accepted (Shimada et al., 2023a; Wikström et al., 2018; Yoshikawa and Shimada, 2015), the critical structural features required by this mechanism, namely, the proton-loading site and a barrier preventing backflow of pumping-protons, have not been experimentally identified (Shimada et al., 2023a; 2023b). In contrast, the H-pathway mechanism proposes that pumping-protons are actively transferred from the N- to the P-side through the H-pathway, while the water-forming protons are delivered via alternative pathways (Tsukihara et al., 2003; Yoshikawa et al., 1998). The H-pathway mechanism of bovine CcO is summarized schematically in Figure 1. The H-pathway is composed of a hydrogen-bond network and a water channel as describe in Figures 1A,C. The hydrogen-bond network is attached to heme *a* via two hydrogen bonds between its formyl and D-ring propionate groups (Figures 1A,D). This structure indicates that pumping-protons on the hydrogen-bond network are expelled to the P-side through the network by electrostatic repulsion generated by the net-positive charges formed on heme *a* upon electron transfer to the O_2_-reduction site. The redox-coupled structural changes illustrated in Figure 1A constitute the active proton release site of bovine CcO (Tsukihara et al., 2003; Yoshikawa and Shimada, 2015). In the oxidized form, pumping-protons are transferred up to D51 at the upper end of the H-pathway. The hydrogen-bond network of the H-pathway includes R38, Y371, a fixed water molecule hydrogen-bonded to one of the propionate groups of heme *a*, the peptide bond between Y440 and S441, and D51 (Figure 1A). Upon reduction of heme *a* (Figure 1A, right), the carboxyl group of D51 migrates from the protein interior to become exposed at the P-side surface (Tsukihara et al., 2003). During this process, protons are relayed through the peptide bond. It has been shown that protons can be transferred through peptide bonds, as schematically represented (Perrin, 1989):

$$H^{+}+–C=O–\text{NH}–\;\to \;–C\left(\text{OH}\right)={\text{NH}}^{+}–\;\to \;–C\left(\text{OH}\right)=N–+H^{+}\;\to \;–C=O–\text{NH}–+H^{+}$$

**FIGURE 2:**
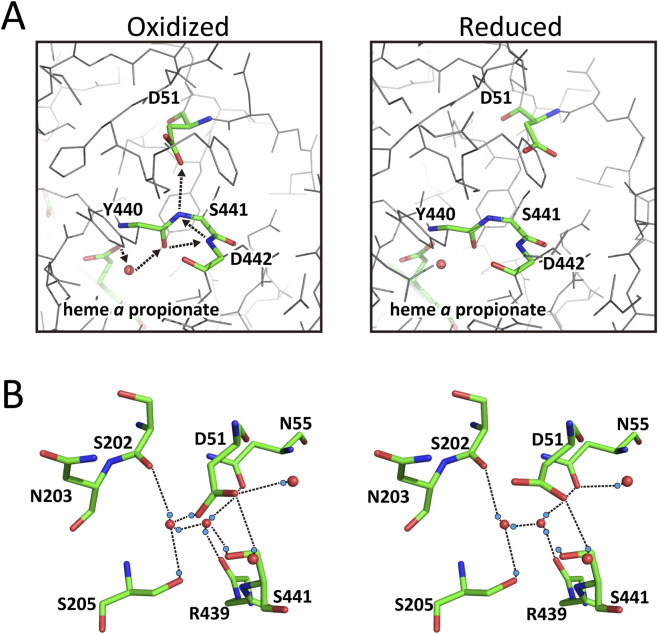
The X-ray structural changes in the P-side end region of the H-pathway of bovine CcO. **(A)** The X-ray structures of part of the hydrogen-bond network of the H-pathway of bovine CcO, from the fixed water (an orange sphere) hydrogen-bonded to one of the two heme *a* propionate groups to D51, in the oxidized (left) and reduced (right) states. The dotted arrows in the left panel indicate the pumping-proton transfer pathway for the multistep transfer, as schematically described in Figure 1B. Upon reduction, D51 migrates towards the molecular surface facing the P-side phase, as shown in the right structure. The structural changes are schematically summarized in Figure 1A. **(B)** The multiple structures of D51 identified in the CO-bound fully reduced bovine CcO crystals. The occupancies of the left and right structures are 80% and 20%, respectively. The red and blue spheres denote the positions of oxygen atoms of fixed water molecules and hydrogen atoms. The dotted lines denote hydrogen bonds. The positions of the hydrogen atoms were deduced from the positions of oxygen atoms of the fixed water molecules and amino acid residues hydrogen-bonded to them. D51 in the left structure is protonated, while it is deprotonated in the right structure.

The intermediate–C(OH) = NH^+^– is referred to as an “imidic acid.” A simulation analysis revealed that the final step of this transfer (–C(OH) = N– → –C=O–NH–), as illustrated by arrow S_3_ in Figure 1B, has the highest energy barrier, rendering the process too slow under physiological conditions (Kamiya et al., 2007). However, an alternative route identified in the X-ray structure of bovine CcO (arrows, S_1_ and S_2_, in Figure 2B) provides a bypass that allows sufficiently rapid proton transfer within the physiological time scale (Kamiya et al., 2007).

The water channel contains several water cavities, depicted as circles or ovals in Figure 1C, each capable of holding at least one water molecule. These cavities facilitate water (and thus proton) movement through the channel by allowing water molecules to move freely. It has been shown that a cavity near the upper end of the water channel disappears upon oxidation (Figure 1C). A redox-coupled 3D structural change identified at a high resolution (Shimada, et al., 2017; Yano et al., 2016) indicates that the open-closed transition of the water cavity is triggered by a translational migration of the heme *a*_3_ plane, which induces a structural change in the side chain of S382 via a hydrophobic interaction between the vinyl group of heme *a*_3_ and L381 as schematically illustrated in Figure 1E. This redox-dependent cavity loss likely halts efficient proton transfer by interrupting water flow through the channel. A preliminary finding of this open–closed transition, driven by redox-coupled structural changes, was reported approximately 2 decades ago based on X-ray structures of oxidized and reduced bovine CcOs at 1.8 Å and 1.9 Å resolution, respectively (Tsukihara et al., 2003). The X-ray structures of the water channels of all intermediate forms of CcO involved in the catalytic cycle of CcO, except for the R-form, show the channel in the closed state (Shimada et al., 2021). This structural feature indicates that, in each catalytic cycle, four pumping protons must be transferred in the R-form through the water channel up to above the closed/open (gating) point of the water channel and retained for sequential donation to the hydrogen-bond network of the H-pathway. The structure of a large water cluster including a Mg^2+^ ion observed in high resolution X-ray structures of bovine heart CcO strongly suggests this pumping-proton storage function (Yano et al., 2016). Thus, the structure suggests that in each of the four proton-pump transitions (Pr→F, F→O, O→E, and E→R), one proton is released (from the water cluster) to the P-side, coupled with the uptake of one water-forming protons from the N-side. However, careful measurements strongly suggest that in each proton-pump transitions, one proton release to the P-side is coupled with two proton uptakes from the N-side (Bloch, et al., 2004; Faxén, et al., 2005; Siletsky et al., 2004). This finding strongly suggests that in each transition, in addition to one water-forming proton, one pumping-proton is collected from the N-side. The pumping-protons are unlikely to be stored in the Mg^2+^ containing water cluster, since the water channel of the H-pathway is closed. Thus, this result strongly suggests that CcO has a pool for storage of these pumping-protons (pumping-proton pool system) below the open/closed point of the water channel. These collected four pump protons are transferred to the Mg^2+^-containing water cluster in the R-state. The existence of such a proton storage (or pool) has been shown experimentally (Zaslavsky, et al., 2004). An extensive search for the pumping-proton pool system is given in this paper.

Using an expression system for bovine heart subunit I in HeLa cells, the function of the H-pathway of bovine heart CcO were examined by mutational analyses (Shimokata et al., 2007; Tsukihara, et al., 2003). Three mutations at the critical residues of the H-pathway, D51N at the pumping-proton exit site, S441P which prohibits imidic acid formation, and V386L/M390W for stopping water movements in the water channel of the H-pathway, resulted in an identical phenotype, abolishment of proton-pump function without affecting the electron-transfer activity (Shimokata et al., 2007; Tsukihara et al., 2003). These mutation analyses strongly confirm the function of the H-pathway proposed based on the X-ray structural analyses as described above.

The A-type bacterial CcOs possess the H-pathway, although some critical residues, such as D51, are not conserved in the bacterial CcOs. Y371F(in bovine number) mutant included in the hydrogen bond network of the H-pathway as described in Figure 1A was as active as the wild type (Lee, et al., 2000). Thus, it was proposed that the hydrogen-bond network is not critically involved in catalytic turnover. However, F371 would introduce a fixed water at the position of the Y371-OH, thereby retaining the hydrogen-bond network. Point mutations of several residues in the water channel of the bacterial H-pathway to less bulkier residues did not show any significant influence on the phenotype function (Lee et al., 2000). These mutations would not be expected to influence the movements of the water molecules in the water channel. Thus, the mutation results for the bacterial H-pathway reported thus far do not provide conclusive evidence disproving the proton pumping function by the bacterial CcOs. The function of the H-pathway of mitochondrial CcO from the yeast, *Saccharomyces cerevisiae*, was examined using a newly developed method for mitochondrial DNA mutagenesis (Maréchal, et al., 2020). The H-pathway mutations, Q411L, Q413L, S382A, S458A, S455A, and S52A (in bovine number), had no significant influence on the enzyme function of yeast CcO, Thus, it was concluded that the H-pathway of yeast CcO is not involved in proton pumping. However, Q411L, Q413L, S458A, and S455A, are located in or near the water channel of the H-pathway. The structural changes in these mutations are too small to block the water channel. These mutations are not able to eliminate the water cavities in the water channel in contrast to the case of the double mutation of bovine CcO, V386L/M390W described above (Shimokata et al., 2007). In bovine CcO, S382 is hydrogen-bonded to the hydoxyfarnesyl ethyl group of heme *a*, eliminating a water cavity of the H-pathway and blocking water access to the hydrogen-bond network of the H-pathway in the oxidized state as described in Figure 1C. However, an energy-minimization analysis indicates that the structural changes in the S382A mutation are too small to eliminate the close/open transition detectable in the wild type enzyme (Shimada et al., 2023a). In other words, S382A substitution is unlikely to induce a water cavity in the oxidized state. S52 is located in a relatively hydrophilic environment facing the P-side phase. The S52A mutation is unlikely to influence the function of the H-pathway. These mutation results are therefore insufficient to disprove the involvement of the yeast H-pathway in the proton pumping. It is noteworthy that, in general, only when a mutation of a residue has a clear effect on the function of a protein can mutational analysis demonstrate the critical involvement of the residue in the function of the protein (Shimada et al., 2023a; Shimada et al., 2023b).

The necessity of protonation of H413 (Figure 1C) in the water channel of the H-pathway of bovine CcO for the incorporation of water molecules into the channel has been proposed based on an atomic molecular dynamic simulation (Sharma et al., 2017). In the X-ray structure of the resting oxidized form at 1.8 Å resolution (PDB ID:1V54), the main part of the water channel is not sufficiently hydrated to support facile proton transfer. The microenvironment of H413 near the entrance of the water channel facing the N-side cannot provide a sufficiently low pH to protonate H413. Thus, it has been proposed that the water channel is not able to transfer protons. However, improved X-ray structures of CcO at 1.55 Å resolution or higher (PDB ID:5B1A, 5B1B, 5ZCQ, 5ZCP, 7COH, 7VUW, 7VVR, and 7YPY) show that the water channel is fully hydrated, yielding a structure identical to that obtained by the above simulation assuming protonated H413, thereby demonstrating that the water channel is active. The 1.8 Å resolution of PDB ID:1V54 is not sufficiently high to resolve the water molecules inside the channel. This issue has been noted previously (Shimada et al., 2018; Shimada et al., 2023a; Supplementary Material of Shimada et al., 2023b).

CcO is present in virtually all aerobic organisms and has evolved structurally to adapt to diverse environmental conditions. Based on evolutionary relationships, CcOs are classified into three families: A, B, and C. The A-family CcOs, including mammalian CcOs, have D- and K-pathways, while B-family CcOs lack an active D-pathway. The A- and B-family CcO conserve the crosslink between Y244 (in bovine number) and one of the three histidine imidazole groups coordinated to Cu_B_. C-family CcOs also lack an active D-pathway and Y244 (in bovine number) is replaced by a tyrosine residue in an adjacent helix. Thus, in C-family CcOs, Y244 is not conserved, but the Y-H covalent bond is conserved (Pereira et al., 2001). As described above, the H^+^/e^−^ is one in A-family CcOs, but 0.5 in B- and C-family CcOs (Han, et al., 2011). The simplest interpretation for the diversity is that, to adapt to environments with low O_2_ concentrations, electron transfer activity is increased by lowering the energy coupling efficiency (the H^+^/e^−^).

A previous study analyzing conserved primary structures (amino acid sequences) of known CcOs from all the three families revealed that only six histidine residues coordinating the redox-active metal centers (low-spin iron, high-spin iron, and Cu_B_) and one tyrosine covalently linked to the imidazole group of a histidine coordinating Cu_B_, are universally conserved. Based on these findings, it was suggested that no specific proton-conducting pathways (such as the D- or H-pathway) are directly responsible for proton pumping. Instead, the conserved metal centers and their coordinating residues are proposed to fulfill the function of CcO, though no explicit pumping mechanism based on these features was presented (Chang et al., 2009). However, it is possible that an identical function is preserved by different amino acid sequences. Therefore, even when the amino acid sequences involved in functional sites differ, functional conservation can be achieved through 3D structural conservation. In fact, the functional similarity of A-family CcO across different organisms has been shown by comparing structural and microscopic electrostatic and thermodynamic properties of the key residues (Popovic et al., 2010). In this study, we investigated the evolutionary conservation of 3D structures related to the H-pathway by analyzing experimentally determined CcO structures from six representative CcO species deposited in the Protein Data Bank (PDB). Our inspection indicates that the key structural components required for the H-pathway mechanism—proton-loading, active proton release, gated collection of pumping protons, and dual proton storage sites for effective proton-pumping—are fundamentally conserved across all three CcO families. Despite notable structural diversity, likely arising from adaptations to different environmental conditions (Han et al., 2011), this structural conservation strongly supports the functionality of the H-pathway in proton pumping. For comparison, we also reviewed the experimental results supporting the D-pathway mechanism and found that, although these results do not disprove the H-pathway mechanism, structures that would prevent pumping-proton leakage to the O_2_ reduction site and to the N-side surface, futures indispensable for the D-pathway to drive proton pumping, have not been identified experimentally.

## 2 Materials and methods

To investigate the structural conservation of the H-pathway across the three CcO families, we examined representative crystal structures. It is known that CcO isolated under aerobic conditions exists in a form distinct from the oxidized state that occurs under turnover conditions; this form is referred to as the “resting oxidized” state (Moody, 1996). Nevertheless, the resting oxidized form has been widely used as a model of the oxidized state (Yoshikawa and Shimada, 2015), and we adopt this convention here. For simplicity, we refer to this state as the “oxidized” form throughout this study. Likewise, the fully reduced form, in which all four redox-active metal sites are reduced, is referred to here as the “reduced” form. The following structures were analyzed: for A-family, oxidized and reduced forms of bovine CcO (PDB ID: 7YPYand PDB IDs: 5B1B and 7EV7, respectively), *S. cerevisiae* (PDB ID: 9ETZ), oxidized and reduced forms of *Rhodobacter sphaeroides* CcOs (PDB IDs: 2GSM and 3FYE), oxidized forms of *Paracoccus denitrificans* wild (PDB ID:3HB3) and N98D mutant CcOs(PDB ID:3EHB), for B-family, *Thermus thermophilus ba*_3_ CcO structures determined from crystals in the absence and presence of a reducing agent, dithionite (PDB IDs: 3S8F and 3EH5), and for C-family, oxidized form of *P. stutzeri cbb*_3_ CcO(PDB ID: 5DJQ). To identify cavities potentially accommodating water molecules, the program VOIDOO (Kleywegt and Jones, 1994) was used with a probe radius of 1.20 Å.

## 3 Results

### 3.1 The structures for pumping-proton loading and unidirectional release to the P-side in the hydrogen bond network of the H-pathway

#### 3.1.1 Bovine CcO

The highest resolution X-ray structures relevant to pumping-proton loading and unidirectional release to the P-side of bovine CcO, from heme *a* propionate to D51, as schematically illustrated in Figure 1A, are shown in Figure 2. The orange sphere in Figure 2A indicates the position of the fixed water molecule bridging the heme *a* propionate and the peptide C=O of Y440. Through the network that includes this fixed water, pumping-protons are transferred to D51 in the oxidized state via the pathway described in Figure 2A (left), following the multistep pathway shown in Figure 1B. Upon reduction, the protonated carboxyl group of D51 migrates toward the P-side surface (Figure 2A, right). Figure 2B shows the X-ray structure (PDB: 7EV7) of the CO-bound fully reduced form of bovine CcO determined at 1.6 Å resolution, which displays two 3D structures of D51. The blue spheres indicate hydrogen atom positions deduced from nearby proton-accepting groups. The left and right structures represent the protonated and deprotonated states of D51, with occupancies of 80% and 20%, respectively. The electron density of the ligand-free fully reduced form confirms this multiple structure in the D51 region.

In the oxidized state, D51 is fully protonated (Figure 1A). Thus, upon reduction of heme *a*, protonated D51 moves toward the protein surface (Figure 2B, right). Upon re-oxidation, after deprotonation of D51 by taking the structure given in Figure 2B right, it returns to its original position (Figure 2A, left) to accept a new pumping-proton via the water channel. This redox-coupled motion effectively prevents proton back-leak from the P-side. Moreover, these structural changes suggest that protons are pumped upon oxidation of heme *a*, consistent with previous experimental results (Capitanio et al., 2000a; Capitanio et al., 2000b).

#### 3.1.2 *R*. *sphaeroides* CcO

**FIGURE 3:**
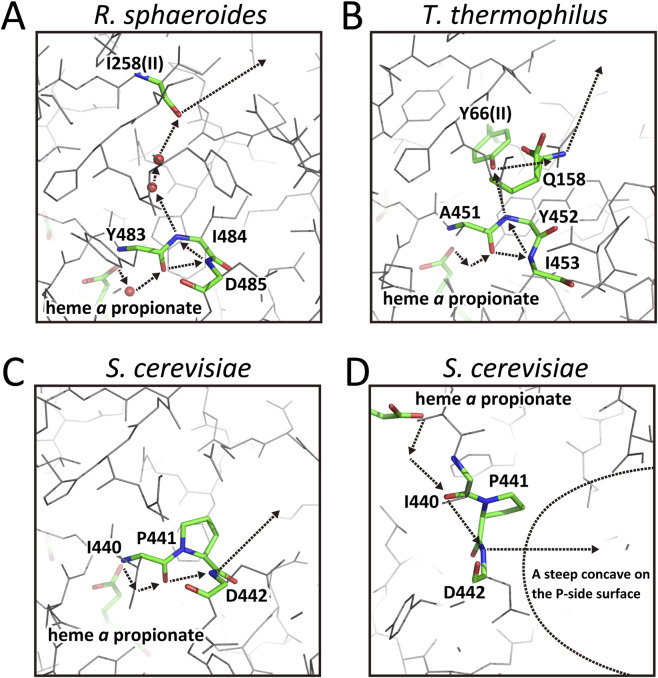
The X-ray structures of the hydrogen-bond networks of the H-pathway of *R*. *sphaeroides*, *T*. *thermophilus, and yeast* CcOs from the fixed water that is hydrogen bonded to the heme *a* propionate to the P-side surface. **(A)** *R. sphaeroides* CcO. The fixed water, hydrogen-bonded to one of the propionates of heme *a* is marked by an orange sphere. The peptide C=O of I258(II) is at the upper end of the hydrogen bond network in the oxidized state. The dotted arrows denote the pathway and direction of the pumping proton transfer. The long arrow shows the pumping proton release site and direction. The 3D structure of the multistep proton transfer pathway through the peptide bond in the network is well conserved, although the residues in the bovine CcO, Y440, D442, S441, and D51 are replaced with Y483, D485, I484, and a fixed water. **(B)** *T. thermophilus* CcO. The fixed water hydrogen-bonded to one of the propionates of heme *a* is not as clear as in that of *R. sphaeroides* CcO shown in panel **(A)**. The structure around the less clear water molecule site strongly suggests the presence of the water. The dotted arrows show the pathway for pumping-proton transfer through the multistep proton transfer, as described in Figure 1B. In this CcO, D51 in bovine CcO is replaced with Y66, while Q158 is at the exit of this proton transfer system, as indicated by a long arrow. **(C)** A cryo-EM structure of the hydrogen-bond network of the H-pathway of yeast (*Saccharomyces cerevisiae*) CcO from the fixed water hydrogen-bonded to one of the propionates of heme *a*. The dotted arrows indicate the position of the pathway. The long arrow shows the location and direction of the pumping-proton release. The 3D structure of the hydrogen-bond network from the propionate to the peptide NH of D442 is well conserved among the A- and B-family CcOs reported here. **(D)** An alternative view of the pumping-proton transfer pathway of yeast CcO from the fixed water hydrogen-bonded to the propionate of heme *a*, showing a steep concavity including the pumping-proton exit. A dotted curve shows the concavity of the molecular surface facing the P-side phase, detectable only in this CcO. The fixed water bridging the heme *a* propionate and the peptide C=O is less clear than in bovine and *R. sphaeroides* CcOs. However, the structure surrounding the water site strongly suggests the presence of the water.

The proton transfer pathway from the heme *a* propionate to D51 observed in bovine CcO (Figure 2A left) is structurally conserved in *R. sphaeroides* CcO, with substitutions of Y440, D442, S441, and D51 by Y483, D485, I484, and a fixed water, respectively (Figure 3A). The fixed water at the position corresponding to D51 in bovine CcO is connected to the P-side surface via another fixed water and the peptide C=O of I258 in subunit II (Figure 3A, dotted arrows). The long dotted arrow indicates the direction and position of proton release to the P-side. The structural similarity between the bovine and *R. sphaeroides* CcOs in the pumping-proton transfer pathway via the peptide bond is evident. Despite the lack of clear redox-coupled structural changes, the hydrogen-bond network from the fixed water (corresponding to D51 in bovine CcO) to the peptide C=O of I258 (Figure 3A) likely prevent spontaneous back-leak of protons. The absence of a stable proton acceptor such as D51 suggests that pumped protons are directly released to the P-side upon oxidation. The wild-type CcO from *P. denitrificans* in the oxidized state exhibits an X-ray structure identical to that shown in Figure 3A (PDB ID:3HB3).

#### 3.1.3 *T*. *thermophiles ba*_3_ CcO

In the oxidized state this CcO also exhibits a hydrogen bond network from the heme *a* propionate to the P-side surface that is sterically identical to that of the bovine CcO, including residues, A451, I453, Y452 and Y66(II), as indicated by dotted arrows (Figure 3B). Although the Asp51 in bovine CcO is replaced by Y66 in this CcO, the close similarity in the 3D structure of the hydrogen bond network from the heme *a* carboxyl group to D51 (bovine) or Y66 (*T*. *thermophiles*) between bovine and this B-family CcO is clear. As described below, the X-ray structure of the reduced form of this CcO has not yet been obtained. However, a facile redox-coupled conformational change of Y66, analogous to that of D51 in the bovine CcO, appear unlikely due to steric hindrance of Q158. Although the hydrogen-bond network from Y66 to the P-side surface is considerably shorter than that in *R. sphaeroides* CcO, the low proton affinity of Q158 may effectively block pumping proton back-leak. Thus, this CcO is likely to release pumping-protons upon oxidation.

#### 3.1.4 Yeast CcO

The 3D structure of *S*. *cerevisiae* CcO within the mitochondrial electron transfer complex was determined by cryo-EM (Berndtsson et al., 2020). As described above, in bovine CcO the imidic-acid intermediate formed between Y440 and S441is proposed to facilitate proton transfer from R38 to D51 as illustrated in Figures 1A,B. In yeast CcO, S441 is replaced by proline (P441), making imidic acid formation—and thus proton transfer through peptide bond—impossible (Figure 3C). Nevertheless, a plausible proton transfer pathway from the heme *a* propionate to the P-side surface exists (Figure 3C, dotted arrows). Instead of imidic-acid formation, proton transfer may occur via the fixed water (hydrogen-bonded to the heme *a* propionate), the peptide C=O of I440, and the peptide NH of D442, which is exposed to a pronounced surface concavity (Figure 3D). This unique concavity is only observed in yeast CcO. Sterically, this peptide NH of D442 in yeast is analogous to the peptide NH of D442 in bovine CcO, which enhances the bypass proton transfer via the S1 and S2 steps in Figure 1B. Thus this NH group in yest CcO also promotes the proton transfer, not to the peptide C=N as in Figure 1B, but to the P-side phase. In yeast, the peptide C=O of I440 is likely a weak proton acceptor, since P441 prevents the imidic-acid formation between I440 and P441. Therefore, I440-C=O may contribute to preventing pumping-proton back-leak. The system for the multiple step proton transfer, as shown in Figure 1B (i.e., Y440-S441-D442) is not fully conserved in yeast. However, the critical group driving the multiple steps given in Figure 1B, the NH of D442 in bovine CcO, is conserved in yeast. In bovine CcO, I440 and P441 in yeast are replaced by Y440 and S441. The S441P mutation in bovine CcO inhibits proton pumping without affecting O_2_ reduction (Shimokata et al., 2007). Unlike yeast CcO, the peptide NH of D442 in bovine CcO is shielded from the P-side, and no concavity is present (Figures 2A,B). Thus, the S441P mutation blocks proton pumping in bovine CcO, as mentioned in Introduction.

#### 3.1.5 *P*. *stutzeri cbb*_3_ CcO

**FIGURE 4:**
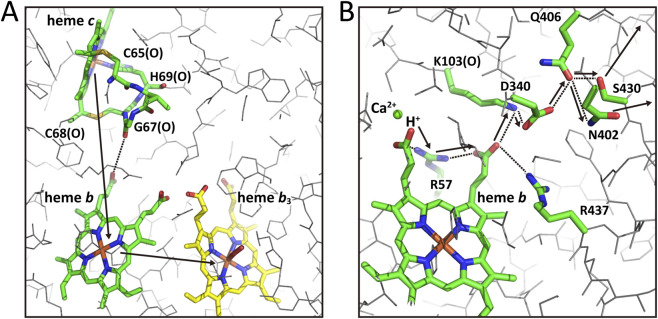
The X-ray structures of electron and proton transfer pathways through the low-spin heme *b* in the C family CcO, the *cbb*_3_ CcO, from *P*. *stutzeri*. **(A)** The location of the three redox-active metal sites, hemes *c*, *b*, and *b*_3_, corresponding to Cu_A_ and hemes *a* and *a*_3_ in bovine CcO. The heme *c* iron is located at the site corresponding to that of Cu_A_ in bovine CcO. The dotted line denotes a possible electron transfer pathway from heme *c* to heme *b* via the peptide C=O and one of the propionates of heme *b*. The two arrows indicate the direction of electron transfer from heme *c* to heme *b*_3_. **(B)** The structure of the hydrogen-bond network of the H-pathway of the *cbb*_3_ CcO for active proton release to the P-side. A green sphere denotes the position of the Ca^2+^ site included in the water cluster for storing pumping protons. The propionate, marked by “H^+^“, is coordinated to the Ca^2+^. The hydrogen-bond network shown by dotted lines illustrates the pathway for pumping-proton transfer from the Ca^2+^-containing water cluster (a closer view for the cluster is given in Figure 7C) to the P-side. The arrows denote the direction of active proton transfer to the P-side surface. The two long arrows denote the position and direction of the pumping proton release at the P-side surface. The three hydrophilic residues with low proton acceptability, N402, Q406, and S430, at the P-side end of the pathway would effectively prevent proton back-leaks from the P-side phase. The pumping protons are transferred to the Ca^2+^containing water cluster from the N-side through the water channel and a hydrogen-bond network including R437 and the two propionates of heme *b,* bridged by R57. The structure from the N-side to Arg437 is given in Figure 6C. Upon oxidation of heme *b*, pumping protons stored in the water cluster including the Ca^2+^ site are transferred to the P-side surface via the hydrogen bond network as indicated by arrows.

The C-family includes only *cbb*_3_-type CcOs, in which Cu_A_, heme *a*, and heme *a*_3_ of A-family CcOs are replaced by heme *c*, heme *b*, and heme *b*_3_, respectively, while Cu_B_ remains at the O_2_ reduction site. To date, the 3D structure of a C-family CcO has been determined only in the oxidized state (Buschmann et al., 2010). In this enzyme, the O_2_ reduction site and heme *b* are located in subunit N, which corresponds to subunit I of the A- and B-family CcOs. Subunit II of the A- and B-families is replaced by subunit O in the *cbb*_3_ CcO, in which heme *c* is located at the position corresponding to the Cu_A_ site in subunit II of A-family CcOs. This enzyme also contains an additional subunit, P, which harbors two heme *c* sites. The heme *c* in subunit O is covalently bound via two thioether bonds with C65 and C68, as illustrated in Figure 4A. The peptide C=O of G67, located between the two cysteine residues, lies 4.5 Å above the propionate carboxyl group of heme *b* (Figure 4A). Furthermore, H69 coordinates the heme *c*. Therefore, electron transfer likely occurs from heme *c* to heme *b* via the peptide C=O of G67 and one of the two propionates of heme *b*, as indicated by a dotted line in the figure. (An alternative, longer pathway, 7.0 Å, for electron transfer from the edge of heme *c* to the heme *b* propionate has been proposed by Buschmann et al., 2010.)

This propionate is connected to another propionate, marked as “H^+^” in Figures 4B, via R57. As shown in Figure 7C, this “H^+^“-marked propionate is coordinated to a Ca^2+^ ion within a water cluster located at a position corresponding to the water cluster above the pumping-proton gate in the H-pathway water channel of A-family CcOs. This propionate forms a hydrogen-bond network extending to the P-side surface, involving R57, the second heme *b* propionate, K103(O), D340, Q406, S430, and N402, as indicated by dotted lines in Figure 4B. These structural features strongly suggest that pumping protons, stored in the Ca^2+^-containing water cluster (*vide infra*), are actively released to the P-side via this hydrogen-bond network upon oxidation of heme *b*, as marked by arrows in Figure 4B. Structural aspects of the proton pathway from the N-side to the Ca^2+^-containing cluster, involving R437, will be discussed in Section 3.2.5. Although this system lacks the peptide NH group mediating the proton active (unidirectional) transfer system seen in the A- and B-family CcOs (Figure 1B), the hydrogen bond network from one of the heme *b* propionates (right side one in Figure 4B) to S430 and N402 is located (conserved) in the region where the proton active release system of the A and B families is located. D340 in the network could function as the loading site for the pumping-protons released from the right side propionate upon oxidation of the heme *b*. The positively charged K103 is likely to prevent backflow of the pumping-proton at D340. These structures, as given in Figure 4B, strongly suggest that the network functions as a proton active transfer system.

The proton active transfer systems in the A, B, and C families are driven by oxidation of hemes *a*, *b,* and *b*, respectively. In spite of significant structural diversity in the hydrogen bond network between the heme propionate and the P-side surface, the X-ray structures of the network strongly suggest that the functions for pumping-proton loading and unidirectional release to the P-side are conserved in all the three families of CcO. This mechanism could be designated as “the low-spin heme driven proton pump”.

### 3.2 The water channel of H-pathway

#### 3.2.1 Bovine CcO

**FIGURE 5:**
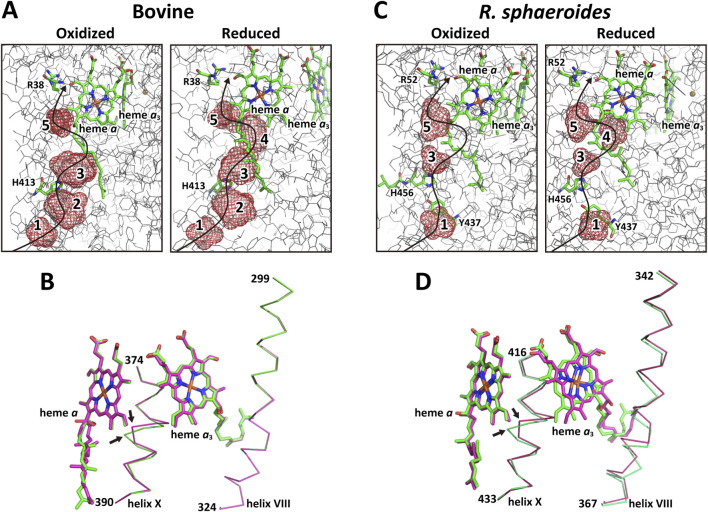
Redox-coupled structural changes in the water channel of the H-pathway. **(A)** Redox-couped structural changes in the water channel region of bovine CcO. The meshes denote the water cavities. Cavity 4 in the reduced state (right) is eliminated upon oxidation (left). H413, located between cavities 2 and 3 is likely to accelerate proton exchange between the two cavities. The curved arrow in each panel shows the possible pumping-proton transfer pathway. **(B)** Redox-coupled structural changes in helix X (marked by two small arrows) and the hydroxyfarnesylethyl group of heme *a*. The structure in the oxidized state (green) is superimposed on that in the reduced state (red). **(C)** Redox-coupled structural changes in the water cavities (red meshes) of *R. sphaeroides* CcO. The cavity corresponding to cavity 2 in bovine CcO is missing in both oxidation states. Y437, found in this CcO, is located at the hydrogen-bond distance of H456, which corresponds to H413 of bovine CcO, as sown in **(A)**. These two residues are likely to facilitate effective proton exchange between cavities 1 and 3. A cavity in *R. sphaeroides* CcO, corresponding to cavity 4 of bovine CcO, is eliminated upon oxidation of the CcO, indicating that the function of the water channel in bovine CcO is conserved in *R. sphaeroides* CcO. **(D)** Redox-coupled structural changes in heme *a*_3_ and helices VIII and X. The green and red structures denote those in the oxidized and reduced states, respectively. The two arrows indicate the redox-coupled structural change in helix X. No redox-coupled structural change in the hydroxyfarnesylethyl group of heme *a* is detectable, while Helix VIII shows a significant redox-coupled shift induced by a significantly large shift in the heme *a*_3_ plane. The helix X shift, triggered by the heme *a*_3_ plane shift upon oxidation, induces the elimination of cavity 4 as shown in **(C)**.

The high-resolution water channel structure (1.5/1.6 Å for the oxidized/reduced forms, respectively) confirms earlier findings (Tsukihara et al., 2003). As shown in Figure 5A, the water channel comprises five water cavities in the reduced state, whereas cavity 4 disappears upon oxidation. This disappearance is triggered by a translational movement of the heme *a*_3_ plane during oxidation, as schematically illustrated in Figure 1E. The heme movement induces a structural change in helix X located near heme *a*_3_, as indicated by the two thick arrows in Figure 5B. H413 bridges cavities 2 and 3, enabling proton transfer between them without water exchange (Figure 5A). The conformational change in the OH group of the hydroxy farnesylethyl side chain of heme *a* is also confirmed at this resolution (Figure 5B). These high-resolution structural results support previous conclusions, as illustrated in Figure 1C (Tsukihara et al., 2003).

#### 3.2.2 *R*. *sphaeroides* CcO

Cavity 2, which is present in the water channel of bovine CcO, is absent in that of *R. sphaeroides* CcO (Figure 5C). However, Y437 in *R. sphaeroides* forms a hydrogen bond with H456, corresponding to H413 in bovine CcO, thereby providing a proton transfer pathway to cavity 3 (Figure 5C). The remaining three cavities (3, 4, and 5) are present in the reduced form of *R. sphaeroides* CcO (Figure 5C, right), and cavity four disappears upon oxidation (Figure 5C, left). This disappearance is triggered by a translational shift of the heme *a*_3_ plane and structural changes in helix X, as shown in Figure 5D. The heme *a*_3_ shift is more pronounced than in bovine CcO, resulting in detectable structural changes in helix VIII in *R. sphaeroides* (Qin et al., 2009), whereas no significant change is observed in the hydroxy farnesylethyl group of heme *a*. R52, hydrogen-bonded to the formyl group of heme *a*, is located at the bottom end of the hydrogen bond network of the H-pathway (Figure 5C). These X-ray structures suggest that the water channel architecture of the H-pathway in bovine CcO is conserved in *R. sphaeroides* CcO in both oxidation states. The water structure of the water channel in *P. denitrificans* CcO in the oxidized state (PDBID:3HB3) was identical to that of *R. sphaeroides*.

#### 3.2.3 Yeast CcO

**FIGURE 6:**
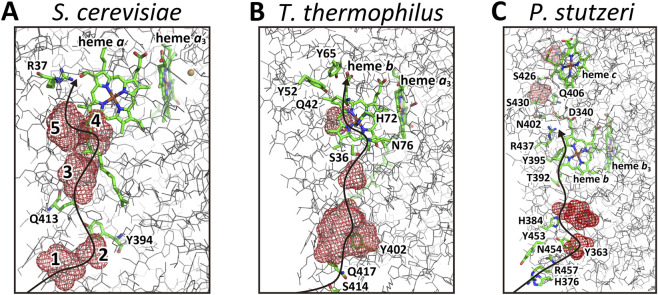
The structure of the water channels of yeast, *T. thermophilis*, and *P. stutzeri*. **(A)** A cryo-EM structure of the water channel region of a yeast (*Saccharomyces cerevisiae*) CcO obtained from its electron transfer complex. Five cavities are detectable and cavities 2 and 3 are connected by Q413 and Y394. R38 in bovine CcO is replaced by R37 as shown in this figure. For the oxidation state of the CcO preparation used for this analysis, see the text (Section 3.2.3 in Results). **(B)** An X-ray structure of the water channel region of the *ba*_3_ CcO from *T. thermophilis* in the oxidized state. Q42 is located at essentially the same site as R38 in bovine CcO. The formyl group of heme *a* in bovine CcO is replaced by a methyl group in heme *b* in the *ba*_3_ CcO. A possible proton transfer pathway, including S36, N76, H72, Q42, Y52, and Y65, is located between the small water cavity and one of the propionates of heme *b*. The large water cluster near the N-side surface corresponds to cavities 1, 2, and 3 of bovine CcO, with Y402, Q417, and S414 at the entrance. **(C)** An X-ray structure of the water channel of the H-pathway of *cbb*_3_ CcO from *P. stutzeri*. Two water cavities (red meshes) are detectable at sites corresponding to cavities 2 and 3 of bovine CcO. They are connected with H384 and Y363, as in *R. sphaeroides* CcO as shown in Figure 5. Several hydrophilic residues form the proton entrance from the N-side to the cavities. Three hydrophilic residues, T392, Y395 and R437, between the upper cavity and the propionate of heme *b,* suggest a possible proton transfer pathway. S430, Q406, and N402 are involved in the pumping-proton release pathway as illustrated in Figure 4B.

The cryo-EM structure of yeast CcO reveals a water channel arrangement closely resembling that of the reduced form of *R. sphaeroides* CcO (Figure 6A). In particular, Q413 and Y394 are likely to facilitate proton exchange between cavities 2 and 3, similar to the H456–Y437 pair in *R. sphaeroides*. R37 is situated at the bottom end of the hydrogen bond network, analogous to R38 in bovine and R52 in *R. sphaeroides* CcOs. The presence of cavity 4 suggests that the water channel is in the reduced form, consistent with the Fe_*a*3_–Cu_B_ distance of 5.1 Å, indicating that the O_2_-reduction site is in the fully reduced state, while helix X appears in its oxidized conformation. These findings suggest that the CcO within the electron transfer complex used for cryo-EM analysis represents a mixture of oxidized and reduced forms. Unfortunately, this structural heterogeneity cannot be assessed, as cryo-EM data lacks the *Fo* data available in X-ray crystallography. Nonetheless, the water channel structure in yeast strongly suggests conservation of the H-pathway channel seen in bovine CcO.

#### 3.2.4 *T*. *thermophilus* CcO

The X-ray structure of oxidized *T. thermophilus* CcO (Figure 6B) shows a large cavity near the N-side surface corresponding to cavities 1, 2, and 3 in bovine CcO, with a smaller cavity, corresponding to cavity 5 in bovine CcO. R38 in bovine CcO, which resides at the bottom end of the hydrogen bond network, is replaced by Q42 in *T. thermophilus*. Unlike bovine CcO, in which R38 is hydrogen-bonded to the formyl group of heme *a*, the corresponding heme in *ba*_3_ CcO is heme *b*, which lacks a formyl group and instead contains a methyl group. As a result, Q42 does not interact with the heme periphery as it does in bovine CcO. Q42 is connected to the small water cavity (above the large cavity corresponding to the cavities 1, 2, and 3 in bovine CcO) via H72, N76, and S36, while the propionate of heme *b* connects to Q42 through two tyrosine residues, Y52 and Y65. These residues form a favorable proton transfer pathway from the water cavity to the propionate group, likely driven by the negative charge generated upon heme *b* reduction. H72, coordinated to Fe_*b*_, replaces R38 in bovine CcO and enables proton collection from the water channel. Protons transiently held at H72 are readily transferred to the propionate of heme *b*, which has higher proton affinity. Upon oxidation, the propionate promotes proton release to the P-side through the pumping-proton release pathway described in section 3.1.3. These results indicate that the proton-pumping function of the water channel is conserved in this B-type CcO.

It has been reported that the X-ray structure of *ba*_3_ CcO crystals reduced with dithionite is essentially identical in the water channel region to that of the oxidized form (Liu et al., 2009; Figure 6B). Absence of a ligand between Fe_*a*3_ and Cu_B_ in the X-ray structure and absorption spectra of hemes *b* and *a*_3_ strongly suggest that the O_2_ reduction site is in the fully reduced state. Therefore, the metal sites of the dithionite-reduced CcO are in the reduced state, while the protein moiety including the water channel of the H-pathway is in the oxidized state. In this crystal structure analysis, a surface-mutated sample, K258R/E4Q (in *T. thermophilus* numbering), was used, which stabilizes the protein moiety strongly in the oxidized structure in the crystal packing (Liu et al., 2007). It is highly likely that the protein moiety in the dithionite-reduced CcO in the crystal is fixed in that of the fully oxidized state by the strong crystal packing constraint. In other words, it is impossible to obtain the protein moiety in the reduced form under this crystal packing using this surface-mutated CcO sample. A cryo-EM analysis of the reduced form in solution is warranted.

#### 3.2.5 *P. stutzeri cbb*_3_ CcO

Cavity analysis reveals two water cavities (brown mesh) near the N-side surface, positioned similarly to cavities 2 (lower) and 3 (upper) in bovine CcO (Figure 6C). These are connected via H384 and Y363, similar to the H456–Y437 arrangement in *R. sphaeroides* (Figure 5C). A potential proton transfer pathway involving T392, Y395, and R437 connects these cavities near the N-side surface with the propionate group of heme *b* (Figure 6C). Y395, T392, and the upper cavity—corresponding to cavity 3 in bovine CcO—are positioned fairly close but not sufficiently close to allow efficient proton transfer. This suggests that redox-coupled structural changes modulate proton transfer at this site, likely forming the proton-pumping gate of the H-pathway in this enzyme. Residues near the N-side surface (H376, R457, N454, and Y363) may facilitate proton uptake. These findings indicate that the functional role of the H-pathway water channel in bovine CcO is conserved in this C-family member. Four residues above R437 in Figure 6C, D340, N402, Q406, and S430, constitute the proton exit pathway, as shown in Figure 4B.

High-resolution structures of the reduced form have been reported only for two A-family CcOs, bovine and *R. sphaeroides* (Qin et al., 2009; Shimada et al., 2023a). To experimentally verify the gating function of the H-pathway water channel, 3D structures in both oxidized and reduced states are required. Particularly for B- and C-family enzymes, such structural data in the reduced state would offer critical insights into the proton-pumping mechanism.

### 3.3 The water cluster for storage of pumping-protons above the pumping-proton gate of the water channel of H-pathway

**FIGURE 7:**
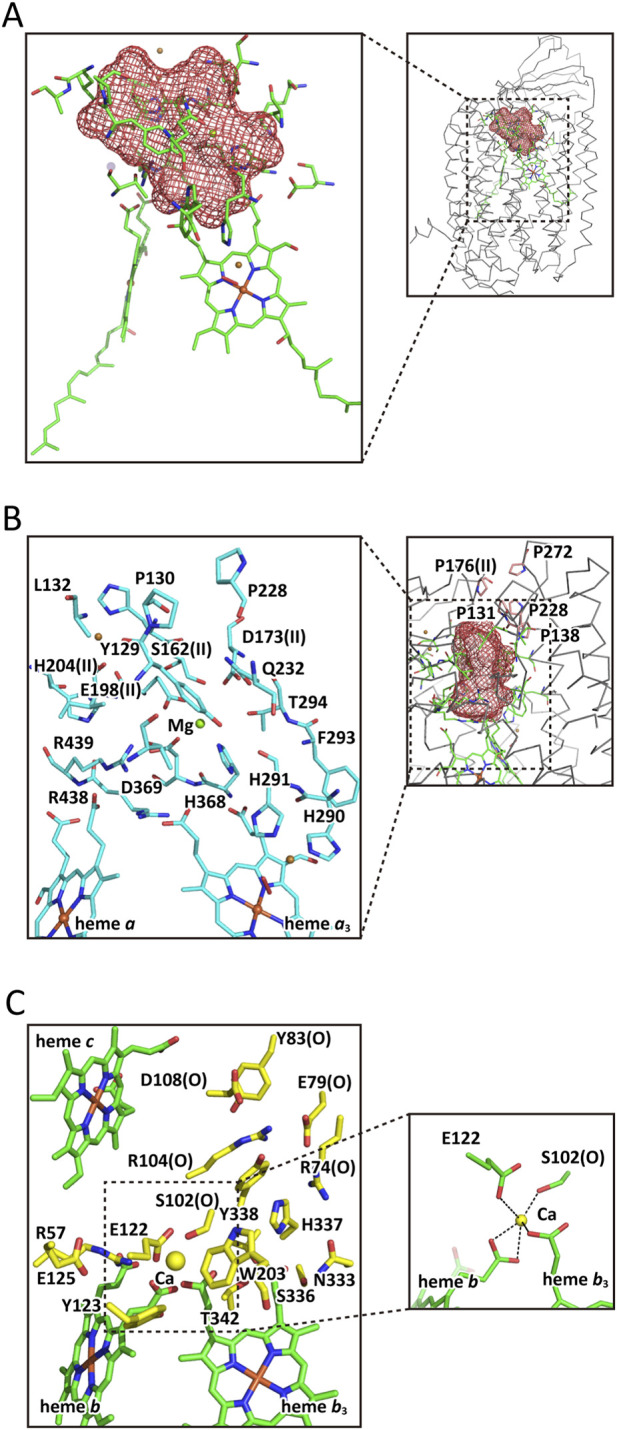
X-ray structures of the water cluster for storage of pumping-protons above the pumping-proton gate of the H-pathway in the oxidized state. **(A)** The shape and size of the water cluster (marked by a red mesh) above the proton gate of the H-pathway of bovine CcO, including the amino acid residues supporting the cluster, shown in green. The inset shows the location of the water cluster in the CcO molecule. **(B)** The amino acid residues involved in the water cluster of the X-ray structure of bovine CcO. A Mg^2+^ site and carboxyl groups of heme propionates are also included in the water cluster. The inset shows the 3D arrangement of the five proline residues (marked by pink structures with residue numbers) in the bovine water cluster near the P-side surface. This arrangement is well conserved in **(A,B)** family CcOs (see text). **(C)** The Ca^2+^-containing water cluster above the pumping-proton gate for pumping-proton storage in *cbb*_3_ CcO from *P*. *stutzeri* in the oxidized state. Yellow structures show the amino acid residues forming the water cluster. The yellow sphere indicates the position of Ca^2+^ in the cluster. The cluster is located at a site corresponding to those of **(A,B)** family CcOs but is more cylindrical in shape. Essentially no homology in the amino acid sequences forming the cluster is detectable compared with those of the **(A,B)** families summarized in Table 1B. The inset shows a magnified view of the Ca^2+^ site.

A large water cluster, including a magnesium ion site, was identified in the X-ray structure of bovine CcO (Yano et al., 2016). The cluster contains approximately 23 water molecules as shown by the brown mesh in Figure 7A. The cluster is located near the P-side surface as shown in the inset. The cluster’s surface facing the P-side is rigidified by a group of five proline residues, effectively preventing water and proton exchange with the P-side phase, as illustrated in Figure 7B inset. These five proline residues are highly conserved among the five A- and B-family CcOs (Table 1A). Although *T. thermophilus* CcO lacks one proline (corresponding to P222 in bovine CcO), four other prolines—P288, P555, and P557 in subunit I, and P55 in subunit II—are located nearby and may compensate for this loss. Figure 7B shows the amino acid residues forming the bovine water cluster, including a magnesium ion coordinated to the carboxyl group of E198. The 3D arrangement of the cluster-forming residues is well conserved among bovine, yeast, *R. sphaeroides*, *P. denitrificans*, and *T. thermophilus* CcOs. This is also true at the sequence level: of the 23 bovine residues, 17, 19, and 14 are conserved in yeast, *R. sphaeroides*, *P. denitrificans* and *T. thermophilus*, respectively (Table 1B). Ten proton-accepting residues (bolded in the table) exist in the bovine cluster and are conserved in yeast, although substitutions such as Q/E, F/Y, and S/D are observed. In *R. sphaeroides* and *P. denitrificans*, all 12 are conserved, with only 1 F/Y substitution. In *T. thermophilus*, two proton-accepting residues (D and E) are replaced with N and Q, respectively, and three additional hydrophilic residues, R, D, and Y, are present. Furthermore, the propionate groups of the two hemes contribute to the cluster as proton-accepting sites. Notably, the *T. thermophilus* cluster lacks a magnesium ion, suggesting that the Mg^2+^ is not essential for the formation or stabilization of the water cluster.

**TABLE 1:** Conservativity in the primary structure of the water cluster of H-pathway.

​	Bovine	*R. sphaeroides*	*P. denitrificans*	Yeast	*T. thermophilus*
A. Proline cluster					
Subunit I	**P130**	**P176**	**P168**	**P131**	**P137**
	**P131**	**P177**	**P169**	**P132**	**P138**
	**P222**	**P266**	**P258**	V223	—
	**P228**	**P272**	**P264**	**P229**	**P221**
Subunit II	**P176**	**P232**	**P196**	**P201**	**P129**
B. Residues in the water cluster, located above the water channel gate, for storing pumping protons delivered from the water channel					
Subunit I	T127	V173	V165	T128	T134
	**Y129**	**Y175**	**Y167**	**Y130**	**Y136**
	P130	P176	P168	P131	P137
	L132	L178	L170	L133	L139
	P228	P272	P264	P229	P221
	Q232	Q276	Q268	**E233**	**R225**
	**H290**	**H333**	**H325**	**H290**	**H282**
	**H291**	**H334**	**H326**	**H291**	**H283**
	F293	**Y336**	**Y328**	**Y293**	F285
	T294	T337	T329	I294	A286
	​	​	​	​	**D287**
	**D364**	**D407**	**D399**	**D364**	**D372**
	**H368**	**H411**	**H403**	**H368**	**H376**
	**D369**	**D412**	**D404**	**D369**	N377
	R438	R481	R473	R438	R449
	R439	R482	R474	R439	R450
Subunit II	L160	I216	I180	I185	I113
	**H161**	**H217**	**H181**	**H186**	**H114**
	S162	S218	S182	**D187**	G115
	**D173**	**D229**	**D193**	**D198**	**E126**
	S197	S253	S217	S222	N150
	**E198**	**E254**	**E218**	**E223**	Q151
	I199	L255	L219	L224	**Y152**
	**H204**	**H260**	**H244**	**H229**	**H157**

No proline cluster is detectable near the water cluster of *P. stutzeri cbb*_3_ CcO.

*T. thermophilus ba*_3_ CcO does not contain Mg^2+^ ion in the water cluster. Residues written in bold letters are proton-acceptable residues. *P. stutzeri cbb*_3_ CcO has no significant conservativity in the amino acid sequence of the water cluster against those of the A- and B-family CcOs.

The X-ray structure of the *P. stutzeri cbb*_3_ CcO reveals that the water cluster above the pumping-proton gate is replaced with a different water cluster containing about 15 proton-accepting and hydrophilic residues, along with a Ca^2+^ ion (yellow structures in Figure 7C). The *cbb*_3_ water cluster is more cylindrical in shape compared to the bovine cluster shown in the red cage in Figure 7A. The Ca^2+^ ion is coordinated to the propionate groups of heme *b* and heme *b*_3_, as well as E122 and S102 in subunit O (Figure 7C, inset). The positive charge of Ca^2+^ likely prevents proton leakage from the cluster by stabilizing the deprotonated states of the propionates. The structural conservation seen in the A- and B-family water clusters is absent in the *cbb*_3_ enzyme. Indeed, the proline cluster that rigidifies the water-cluster in the A/B families is lacking in *P. stutzeri* (Table 1A). However, the cluster is still insulated from the P-side phase by the surrounding protein, including subunit P. Thus, the structure is highly likely to function as a pumping-proton storage site, similar to the water cluster above the pumping-proton gate in A- and B-family CcOs.

### 3.4 Pumping-proton pool systems from the N-side phase below the pumping-proton gate of the water-channel

**FIGURE 8:**
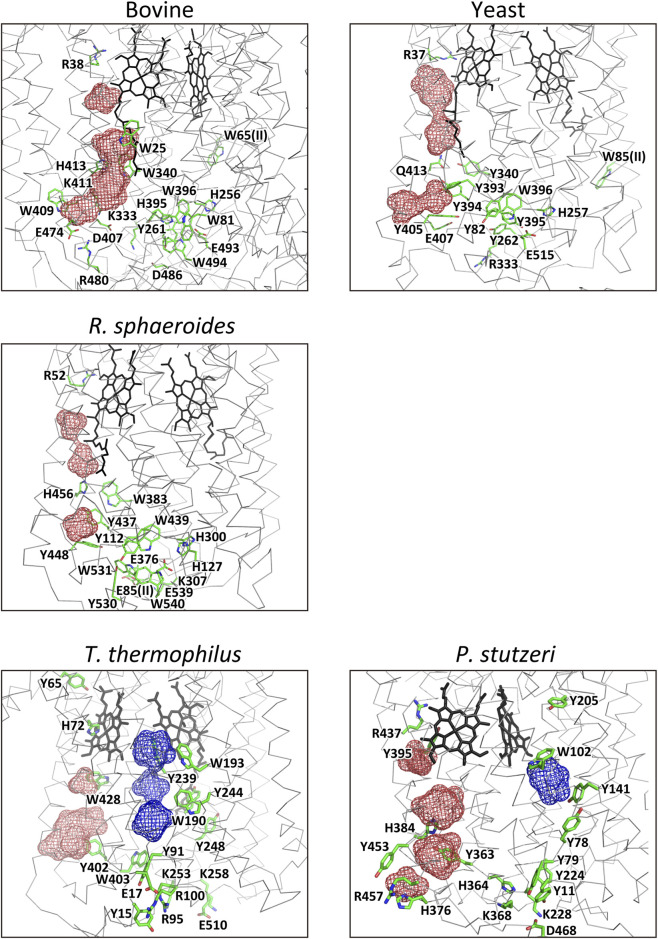
The pumping-proton pool systems below the pumping-proton gate of the water channel. The proton accepting residues below the level of the four carboxyl groups of the propionates of the hemes *a* (*b*) and *a*_3_ (*b*_3_) and the magenta and blue meshes, showing the water accessible spaces of H and D pathways, respectively, are illustrated for CcOs of bovine, yeast (*Saccharomyces cerevisiae*), *R. sphaeroides*, *T. thermophilus ba*_*3*_, and *P*. *stutzeri cbb*_3_. Since the D-pathway in the A-family CcOs, which transfers the water-forming protons, is unable to retain the pumping protons, the D-pathway of bovine, yeast and *R. spharoides* CcOs is not included in the pumping proton storage system. The blue meshes for D-pathways do not include the space connecting the P-side end of the D-pathway and the O_2-_reduction site. This space is highly hydrophobic and is used as the O_2_-transfer pathway under turnover conditions.

Numerous proton-accepting amino acid residues are located in the N-side region below the pumping-proton gate of the H-pathway water channel, as shown in Figure 8. These residues, along with the water cavities of H-pathways, likely contribute to the temporary storage of pumping protons. As the H-pathway channel opens only in the R-state, the H-pathway could serve as a trap for pumping protons during the four-step catalytic cycle. The H-pathway, together with the proton-accepting residues in the A-family CcOs, as illustrated in Figure 8, appears to provide sufficient capacity for storing pumping protons: four proton equivalents per catalytic cycle. In the B- and C-family CcO, D-pathway, which does not transport water-forming protons, may also serve as the pumping proton trap, as illustrated in Figure 8 (Han et al., 2011). The X-ray structure of the pool system of *P. denitrificans* identical to that of *R. sphaeroides* is not given in Figure 8.

## 4 Discussion

### 4.1 Evolutionary conservation of 3D structures essential for the H-Pathway mechanism

Our structural analysis of representative CcOs reveals that the 3D features essential for proton pumping via the H-pathway mechanism are evolutionarily conserved across all three CcO families. These conserved features include.

1. The system for loading and actively releasing pumping protons to the P-side,
2. The water channel with a gate that opens for the collection of pumping-protons from the N-side before the catalytic cycle starts and closes during the catalytic cycle to prevent pumping-proton leakage,
3. The water cluster above the pumping-proton gate for pumping-proton storage, and
4. The pumping-proton pool system, located below the pumping-proton gate, which enables the facile supply of pumping protons to the water cluster when the gate opens.

This structural conservation strongly supports the hypothesis that the H-pathway is the functional proton pump. Except for the extensively studied bovine CcO, previous structural studies have not focused on identifying these H-pathway-related elements. Therefore, the structural data presented here, excluding that of bovine CcO, constitute the first such analyses, even though the corresponding crystallographic datasets have long been available in the PDB.

### 4.2 Comparison of the D-pathway mechanism with the H-pathway mechanism

#### 4.2.1 Experimental findings supporting the D-pathway mechanism

##### 4.2.1.1 Mutational analyses for the oxidative phase

X-ray structural and site directed mutagenesis analyses identified two possible proton-transfer pathways, connecting the N-side to the O_2_ reduction site (Fetter, et al., 1995; Hosler et al., 1996). One of the pathways contains E242 and D91 (D-pathway), whereas the other, contains K319 and T316 (K-pathway). (In Section 4.2., bovine numbering is used. Corresponding residue numbers in the other CcOs are given in Table 2.). The effects of mutations of these protonatable residues within these pathways in a bacterial CcO on the proton and electron transfer activities inside the CcO molecule during the oxidative phase were analyzed by following time resolved absorption spectral changes when the fully reduced enzyme in a detergent-solubilized state was oxidized by an excess amount of O_2_. The proton uptake during the oxidative phase of the enzyme was monitored by absorption spectral changes of a pH-indicator dye (Adelroth, et al., 1997; 1998). A D-pathway mutation, E242Q, completely blocked electron transfer reactions in the oxidative phase (Pr → F and F → O). The lack of the proton uptake coupled with each reaction step was also confirmed by the pH indicator dye. On the other hand, K-pathway mutants, K319M and T316A showed no significant effects on either the electron-transfer reactions or the proton uptake. The results indicate that D-pathway, not K-pathway, transfers the water forming protons indispensable for the two steps in the oxidative phase. Under the experimental conditions (i.e., in the detergent-solubilized enzyme and not in the liposome), it is impossible to examine the proton pumping process.

**TABLE 2:** Amino acid residues of D-pathway, located sterically equivalent sites.

Bovine	*R. sphaeroides*	*P. denitrificans*	*T.thermophilus*	*P. stutzeri*
Y19	Y33	Y35	F24	F17
M71	I112	I104	Q82	G70
N80	N121	N113	V80	Y78
D91	D132	D124	R100	L89
N98	N139	N131	M106	A95
N163	N207	N199	I162	V144
G239	G283	G275	G232	G206
E242	E286	E278	I235	A209
H291	H334	H326	H283	H258
T316	T359	T351	S309	S283
K319	K362	K354	T312	G286

The D-pathways of *T. thermoplilus* and *P. stutzeri* CcOs are inactive.

Using a single electron injection technique, one of the oxidative reaction steps, F → O, was examined for various mutant CcO, following vectorial movements of both water-forming and pumping protons inside the CcO molecules incorporated in liposomes (Konstantinov et al., 1997; Siletsky and Konstantinov, 2012; Vygodina et al., 1998). In the wild type CcO, two vectorial proton (positive charge) movements are detected, corresponding to the uptake of the water-forming protons from the N-side and the release of pumping protons to the P-side. Both D-pathway mutants, E242Q and D91N, essentially eliminated these two proton movements, whereas K319M and T316A strains showed no significant perturbations to these proton movements detectable in the wild type enzyme. These results strongly suggest that D-pathway, not the K-pathway, transfers both the water forming and pumping protons in the oxidative phase.

##### 4.2.1.2 Mutational analyses for the reductive phase

When the oxidized CcO is reduced with an excess amount of a reducing agent, such as dithionite, under anaerobic conditions, electrons are transferred from the reducing agent to the O_2_ reduction site, as follows, Cu_A_→ Fe_*a*_ →Fe_*a*3_ →Cu_B_, until all the four metal sites are reduced (fully reduced state). It has been shown that, when dithionite is used as the reducing agent, the electron transfer step, Fe_*a*_ →Fe_*a*3,_ is the slowest (rate determining) step in the above electron transfer from Cu_A_ to Cu_B_. In other words, the Cu_A_→ Fe_*a*_ step is much faster than the Fe_*a*_ →Fe_*a*3_ step, so that Fe_*a*3_ reduction occurs slowly after the complete reduction of Fe_*a*_. In the fast phase, both heme *a* and Cu_A_ are reduced, while in the slow phase the rest of the two metal sites, heme *a*_3_ and Cu_B_, are reduced. Thus, the slow phase corresponds to the reductive phase of the catalytic cycle. Thus, the Fe_*a*3_ reduction, which induces O→E and E→R transitions (the reductive phase), can be followed without using any specialized apparatus for rapid absorbance spectral measurements. Effects of mutations on the reductive phase were examined by measuring absorption spectral changes due to reduction of heme *a*_3_ with dithionite under anaerobic conditions as described above (Wikström et al., 2000). The extent and rate of heme *a* reduction was essentially insensitive to mutations of both D and K-pathways. However, K-pathway mutation, K319M, essentially blocked the reduction of heme *a*_3_. This result indicates that K-pathway impedes the water-forming proton transfer required for the O→E transition. Namely, the water-forming protons necessary for the O→E transition are transferred through the K-pathway. However, both D-pathway mutants, D91N and E242Q, showed clear heme *a*_3_ reduction in the slow phase. However, the extent of the heme *a*_3_ reduction was only about 50% of that detectable in the wild type. The result indicates that, in these D-pathway mutants, the water forming protons for O→E transition are transferred through the K-pathway, while those for E→R transition, through D-pathway. In these mutants, the electrons from heme *a* to heme *a*_3_, leadig the O→E transition, equilibrated between heme *a*_3_ and Cu_B_, so that approximately 50% of heme *a*_3_ becomes reduced. This results has been confirmed by a single electron reduction analysis using a laser flash technique for O→E and E→R transitions. Indeed, the O→E transition is not influenced by the D-pathway mutations, while K-pathway mutant blocked the transition. On the other hand, E→R transition was not influenced by K-pathway mutation but D-pathway mutants block the transition. (Ruitenberg, et al., 2000). These results strongly suggest that at least one proton out of eight protons taken from a single catalytic cycle of CcO, is taken up from K-pathway. Pumping protons in the O→E transition, are likely to be transferred through D-pathway, since K-pathway does not have the proton loading site. Namely, D-pathway could transfer seven protons. Except for the water-forming proton for the O→E transition, both water forming and proton pumping protons are transferred through D-pathway.

Certain decoupled mutants in the D-pathway, such as N98D, N98T, and N98S, retain normal O_2_-reduction activity but lack proton-pumping function. These results are cited as strong experimental results proving that the D-pathway transfers both water-forming and pumping protons (Hosler et el., 1996b, Namslauer et al., 2003; Dürr et al., 2008; Lepp et al., 2008a, Pawate et al., 2002). Using a decoupled mutant of the D-pathway, amplitudes of water-forming proton transfer during the two transitions in the oxidative phase were determined (Lepp, et al., 2008a).

##### 4.2.1.3 Spectroscopic and electrometric analyses for the oxidative phase of bacterial A-family CcOs

The oxidative phase of a bacterial (*P. denitrificans*) CcO reaction during oxidation of the fully reduced CcO after flash photolysis of fully reduced CcO in the presence of an excess amount of O_2_ was examined by highly sensitive visible and ATR-FTIR spectroscopic and electrometric analyses for the enzyme molecules embedded in liposomes (Belevich et al., 2010; Gorbikova et al., 2007; Lepp et al., 2008b). The reaction progress and charge movements perpendicular to the membrane plane inside the CcO molecules were followed by time-resolved spectroscopic and electrometric measurements, respectively, for various mutant enzymes.

**FIGURE 9:**
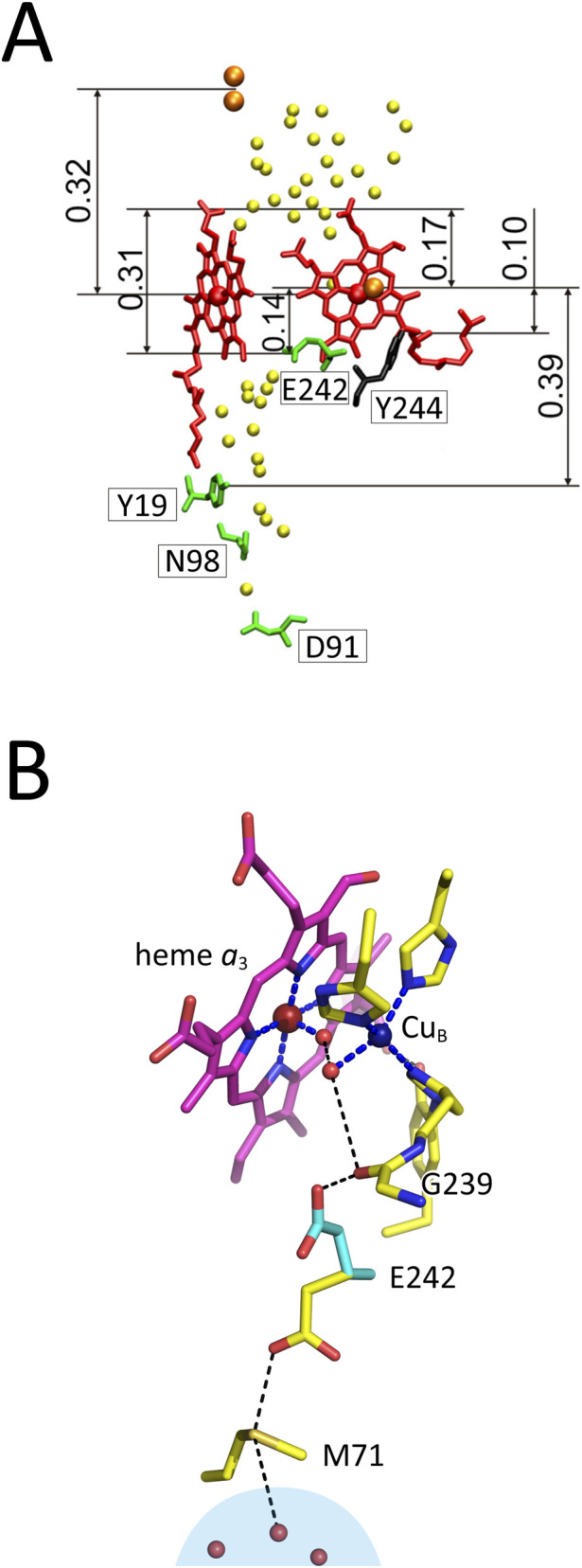
The X-ray structure of the D-pathway. **(A)** The key redox centers and protonatable groups and their relative dielectric depths. Reprinted with permission from (Belevich, et al., 2010). **(B)** The possible 3D structural changes in the E242 side chain in the oxidized bovine CcO. The blue structure shows a hypothetical conformational change estimated from the space surrounding the E242 side chain. Reprinted with permission from (Shimada et al., 2023a).

It has been shown that a positive charge movement in the oxidative phase, determined for a decoupled *R. sphaeroides* CcO species mutated at N98T in the D-pathway, is assignable to that due to a proton transfer from N-side to the O_2_ reduction site (Lepp et al., 2008a). This result has been regarded as an experimental evidence showing that D-pathway transfers water-forming protons. The D91N/Y19F mutant of *P. denitrificans* CcO showed only the R→A→Pr transition, since both the Pr→F and F→O transitions are blocked (or extremely slowed down) by inhibition of water forming proton transfers driving these transitions (Belevich et al., 2010). The charge movement detectable during the transition is assignable to the A→Pr transition, since the R→A transition is not electrogenic. The charge movement during the process corresponds to 41% of that induced by movement of a single positive charge perpendicularly across the CcO molecule from the N-side surface to the P-side surface (designated as *d* in this paper) (Belevich et al., 2010). During the A→Pr transition, a single proton is transferred from Y244 to Cu_B_, A [Fe_*a*_^2+^, Fe_*a*3_^2+^-O_2_, Cu_B_^1+^, Y244OH] → Pr [Fe_*a*_^3+^, Fe_*a*3_^4+^ = O^2-^, Cu_B_^2+^-OH^-^, Y244O^−^] (Belevich et al., 2010). The relative location between Cu_B_ and Y244OH suggests that 0.1 *d* out of 0.41 *d* is induced by the proton translocation (Figure 9). The rest of the charge movement, 0.31 *d*, is consistent to that induced by a single positive charge movement from E242 to a propionate group of heme *a*_3_, which locates near the putative proton-loading site of pumping protons (Figure 9A). Furthermore, E242Q mutant shows no charge movement except for that assignable to the proton translocation from Y244 to Fe_*a*3_ (Belevich et al., 2006, Supplementary Material). This result confirms the above assignment of the 0.31 *d* charge movement to that from E242 to the possible proton loading site. In fact, Q242 residue is not able to hold any proton. However this mutant shows A→ Pr transition induced by heme *a* oxidation, suggesting that heme *a* oxidation is not coupled with the proton pumping, in contrast to the H-pathway mechanism proposal given in Results.

It has been shown that a D91N mutant shows the oxidative phase transition until formation of the F-form, (namely, A→Pr and Pr→F) (Belevich et al., 2010; Gorbikova, et al., 2007). The D91N mutation blocks uptake of water-forming protons from the N-side. Thus, the water forming protons to drive the Pr→F transition in the D91N mutant must be stored between E242 and D91. However, E242 has donated protons to the proton-loading site during the A→Pr transition for proton pumping, as described above. The elimination of the Pr→F transition by Y19F mutation in addition to D91N mutation suggests that Y19 retains the water forming protons for the Pr→F transition. The charge movement assignable to the Pr→F transition is consistent with the proton movement from Y19 to the Fe_*a*3_ iron atom in the high resolution X-ray structure (Figure 9A), indicating that the water forming protons driving the Pr→F transition are also transferred through the D-pathway (Belevich et al., 2010). A time-resolved ATR-FTIR analysis was conducted to confirm the role of Y19 as a water-forming proton donor as described in Supplementary Material.

The above spectrophotometric and electrometric analyses strongly suggest that the D-pathway transfers both the pumping and water-forming protons in the A→Pr and Pr→F transitions, respectively. Furthermore the D-pathway mechanism proposed above has been confirmed by simulation analyses (Sugitani et al., 2008, Saura et al., 2022, Leontyev and Stuchebrukhov, 2009).

#### 4.2.2 Alternative interpretations for the results supporting the D-pathway mechanism and findings that do not conclusively support the D-pathway mechanism

##### 4.2.2.1 Alternative interpretations of the mutation results supporting the D-pathway mechanism

From various mutational results given in Sections 4.2.1.1 and 4.2.1.2, it has been concluded that the D-pathway transfers both pumping and water-forming protons. However, these mutation results do not identify the location of the pumping-proton pathway. An early article on mutation studies states, “Assuming that only two pathways are used for proton uptake, the results from this study indicate that all protons are taken up through the pathway containing E242” (Adelroth et al., 1997). The possibility that the D-pathway is tightly coupled with a pathway for pumping-proton transfer has not been excluded experimentally. Most of these conclusions have been derived while ignoring the possibility. The X-ray structures presented in the Results section provide an alternative interpretation, suggesting that the pumping protons are transferred through the H-pathway, which is coupled with the D-pathway. Furthermore, the fact that a single mutation in the K-pathway, such as K319M, blocks the D-pathway, resulting in complete inhibition and *vice versa*, indicates existence of a tight interaction between the two pathways. This point should not be ignored.

##### 4.2.2.2 The X-ray structure of the space between E242 and the O_2_ reduction site

High resolution X-ray structures of bovine CcO show that E242 is located at the wall of the O_2_ channel which connects the surface of the subunit III in the transmembrane region to the O_2_ reduction site, parallel to the membrane surface. X-ray structures of CcO reported thus far show that the O_2_ transfer channel is composed of highly hydrophobic amino acid side chains and phospholipid tails, which strongly disfavors the presence of hydrophilic molecules including water molecules within it (Yano et al., 2016). Any water wire present would also likely hinder efficient O_2_ transfer. Therefore, under catalytic turnover conditions, the formation and clearance of such water wires from the deeply buried hydrophobic space would be energetically costly and implausible within the timescale of the catalytic turnover of this enzyme. Thus, the proton transfer through the hydrophobic space is highly unlikely. Although the water-wire formation in this space has been proposed by simulation analyses (Liang et al., 2016; Wikström et al., 2003; Zheng et al., 2003), no experimental confirmation for this water-wire formation has been reported. The space around E242 side chain suggests a significant structural flexibility that allows the formation of a hydrogen bond network from the E242 carboxyl group to Fe_*a*3_ as illustrated in Figure 9B (Shimada et al., 2023a). The X-ray structural finding suggests that protons for water formation necessary in the three steps, Pr→F, F→O, and E→R, are transferred through this network, without the formation of any water wire. (These three steps are blocked by D-pathway mutations.) However, pumping-proton transfer from E242 to the putative pumping-proton loading site near the heme *a*_3_ propionate through the hydrophobic space by forming water wires is unlikely to occur.

##### 4.2.2.3 The function of E242

**FIGURE 10:**
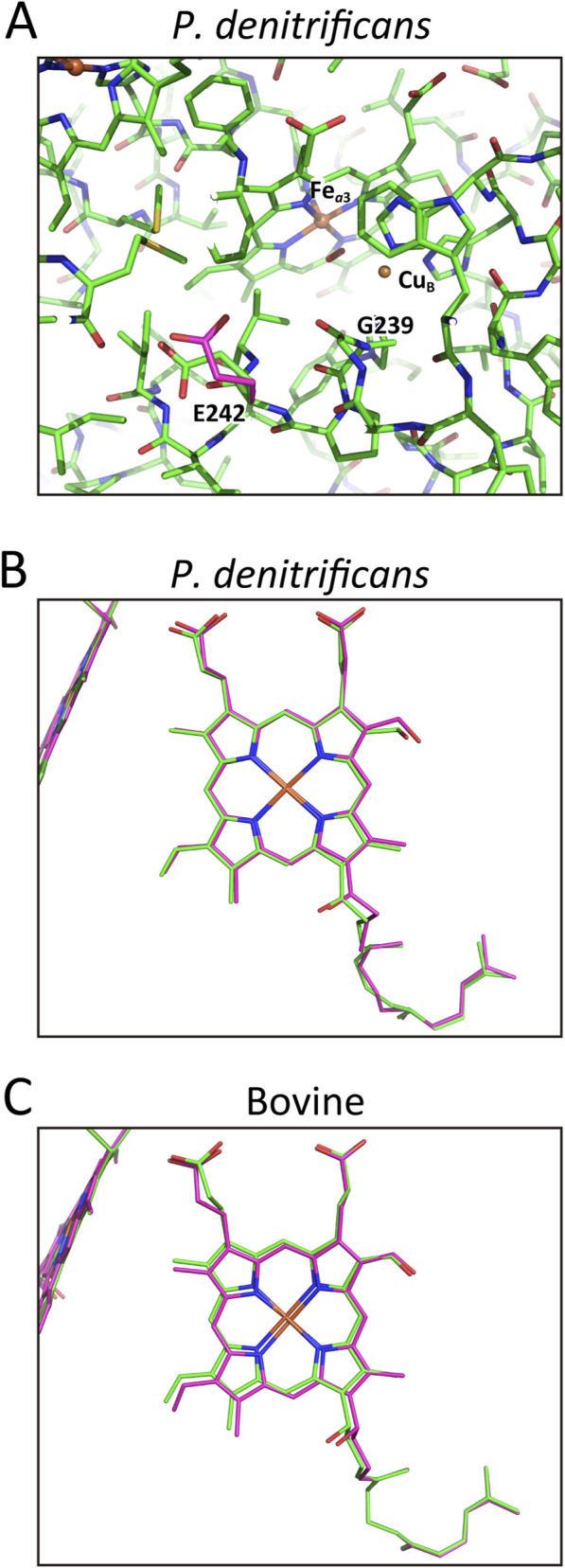
The structures of E242 and heme *a*_3_ of the N98D mutant of *P. denitrificans.* **(A)** Multiple structure of E242 in N98D mutant CcO. The E242 side chain shows two structures, in green and pink, with equal occupancy. The green structure is essentially identical to that of the wild type, while the pink one locates closer to G239 C=O group. **(B)** The structure of heme *a*_3_ plane of the oxidized N98D mutant CcO (red) superimposed on that of the oxidized wild type CcO (green), showing a translational movement upon the mutation. **(C)** The translational movement of heme *a*_3_ plane of bovine CcO upon reduction. The green and red structures show those of the oxidized and reduced forms respectively.

The location of E242 suggests that the side chain is highly flexible enough to form a hydrogen bond network with the O_2_ reduction site as illustrated in Figure 9B. This flexibility suggests that strong interactions exist between E242 and heme *a*_3_. As described above, the E242Q mutation eliminates the electronic charge movement assignable to the proton movement from E242 to the putative pumping-proton loading site near the propionate of heme *a*_3_ as illustrated in Figure 9A. On the other hand, a decoupling mutant, N98D, blocks the proton pump without influencing the transfer of the water forming protons (the O_2_-reduction activity). If the E242Q mutation blocks the pumping-proton transfer simply by removing the COOH group of E242, how does N98D mutant CcO, which has the unmodified E242, block the pumping-proton transfer? In fact, the X-ray structure of the mutant of *P. denitrificans* shows no structure likely to block pumping proton transfer from E242 to the putative pumping-proton loading site. However, a multiple structure is detectable in the E242 side chain of the N98D mutant, composed of one identical to that of the wild type and another located closer to the peptide group of G239 as illustrated in Figure 10A (Dürr et al., 2008). It has been shown that the E242 side chain in the X-ray structure has enough space to form a hydrogen bond with the peptide C=O group of G239 as illustrated in Figure 9B. One of the side chain structures detectable only in the N98D structure is located not close enough to form a typical hydrogen bond with G239 as illustrated in Figure 9B. However, considering the hydrophobic environment of these residues, structural changes in E242 induced by the N98D mutation are highly likely to significantly increase the electrostatic interactions between E242 and G239. Although an X-ray structure of E242Q has not been reported, the structural changes in E242 upon E242Q mutation are likely to be significantly larger than those upon N98D, providing higher electrostatic (or structural) influence on G239.

Interaction between E242 and heme *a*_3_ is directly detectable by comparison of the X-ray structure of the N98D mutant of *P. denitrificans* with that of the wild type, as follows; the N98D mutant molecule of *P. denitrificans* CcO was aligned with that of the wild-type CcO by superposing main-chain atoms of the mutant CcO on the corresponding atoms of the wild-type. The average deviations between the mutant and the wild-type CcO were estimated for heme *a* as 0.16 Å and heme *a*_3_ as 0.28 Å, respectively. The superposed structure of heme *a*_3_ of N98D mutant and the wild-type *P. denitrificans* CcO is shown in Figure 10B, and that of the oxidized and reduced forms of bovine CcO is drawn in Figure 10C. The movement of heme *a*_3_ of N98D mutant is larger than that of heme *a* in the same molecule, indicating that the E242 change induces a selective migration of heme *a*_3_. The shift of heme *a*_3_ of the mutant from that of the wild type (Figure 10B) is smaller than that of the reduced form of bovine CcO from that of the oxidized form (Figure 10C). However, the direction of the shift of heme *a*_3_ by the mutation of *P. denitrificans* CcO is the same as that of the reduction of the bovine CcO. This result indicates that the N98D mutant, which is in the oxidized state, induces a heme *a*_3_ shift similar to but smaller than that detectable upon reduction of heme *a*_3_. As mentioned in Figures 1A, E a translational movement of heme *a*_3_ upon its oxidation induces an open-to-closed transition of the water channel of the H-pathway mediated by helix-X movement (Shimada, et al., 2017; Yano et al., 2016). Thus, it is possible that N98D mutation stabilizes the open state during the catalytic cycle and thereby collapses the proton gradient. Namely, N98D mutant in the A form has no proton to be pumped in the water cluster for storage of pumping-protons above the water-channel gate, giving a decoupled state. Furthermore, via the above interaction system including E242, G239, and heme *a*_3_, Q242 could provide a stronger influence to G239 than that exerted by E242 of the N98D mutant.

**FIGURE 11:**
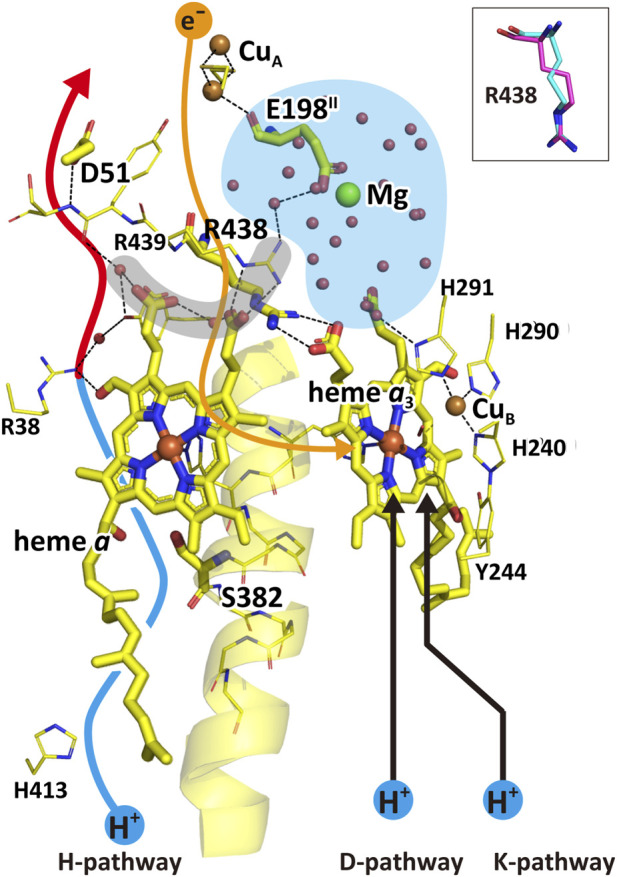
The X-ray structure of the short hydrogen bond network connecting the Mg^2+^-containing water cluster and the hydrogen bond network of the H-pathway, marked by a gray area. The orange arrow indicates the location of the electron transfer pathway from the P-side to heme *a*_3_. One of the heme *a*_3_ propionate groups forms a salt bridge with R438 which shows a redox-coupled structural change as shown in the inset. These structures suggest that heme *a*_3_ controls the function of the short hydrogen-bond network. Reprinted with permission from (Yano et al., 2016).

As illustrated in Figure 11, pumping protons stored in the water cluster for pumping-proton storage above the water channel gate are transferred to the heme *a* propionate through a short hydrogen bond network. The transferred proton at the heme *a* propionate is pumped to the proton loading site above the peptide bond in the hydrogen bond network of the H-pathway upon heme *a* oxidation as mentioned in Results. One of the propionate of heme *a*_3_ is salt-bridged to R438, the side chain of which contacts tightly with R439 included in the short hydrogen bond network and shows redox-coupled structural changes as illustrated in the inset of Figure 11. This redox coupled structural changes suggest that heme *a*_3_ controls the proton transfer through the short hydrogen-bond network. Thus, the N98D and E242Q mutations are likely to influence the proton transfer function of the short hydrogen-bond network via heme *a*_3_ and R438 and thereby weaken or abolish the proton-pumping function.

These results suggest that E242 does not transfer pumping-protons but rather controls the function of heme *a*_3_, which is critical for proton pumping through the H-pathway. These mutation effects are unlikely to perturb the redox potential of heme *a*, which is consistent with the experimental result that the E242Q mutation does not inhibit heme *a* oxidation.

##### 4.2.2.4 Alternative interpretations for time-resolved spectroscopic and electrometric analyses for bacterial A-family CcOs

As described in Section 4.2.1.3, the electrometric analyses of the A→ Pr transition of the *P. denitrificans* CcO showed a charge movement assignable to the proton transfer from E242 to the putative proton loading site, and the charge movement was eliminated by the E242Q mutation without influencing the A→ Pr transition. However, the X-ray structural examinations of the H-pathway, as described in Results, indicate that this charge movement is assignable to that due to the proton transfer through the hydrogen bond network of the H-pathway, triggered by heme *a* oxidation, and to proton collection from the N-side to the proton pool system below the water channel gate (Figure 8). Furthermore, the E242Q mutation is highly likely to decouple proton pumping from heme *a* oxidation, as described in the preceding section (Belevich et al., 2010).

As described in Section 4.2.1.3, the D91N/Y19F and D91N mutation results suggest that the protonated E242 in the A state transfers a proton to the proton-loading site during the A→Pr transition, and that since E242 is deprotonated in the Pr state, Y19 provides the water-forming proton for the Pr→F transition. The proton transfer from Y19 to Fe_*a*3_ was confirmed by the electrometric analysis. These results suggest that D-pathway transfers both pumping and water-forming protons. However, assuming the H-pathway mechanism, an alternative interpretation for these results is possible as follows; E242 donates the water-forming proton for the Pr → F transition while the pumping protons are transferred through the H-pathway, whereas the Y19F mutation blocks the Pr→F transition but does not hold water-forming proton. The positive charge movement induced by the proton transfer from E242 to Fe_*a*3_ is significantly smaller than that from Y19. However, the X-ray structure of the proton-pool system (Figure 8) shows many candidate amino acid residues which could provide the experimentally determined charge movement coupled with the Pr → F transition. Thus, the electrometric charge analysis for these mutants given in Section 4.2.1.3 (Belevich et al., 2010) do not disprove the H-pathway mechanism.

The experimental results obtained by a time-resolved ATR-FTIR analysis, conducted to confirm the role of Y19 as a water-forming proton donor, supports the D-pathway mechanism. However, an alternative interpretation based on the H-pathway mechanism for the ATR-FTIR data is possible, as described in Supplementary Material. In other words, the ATR-FTIR results do not conclusively support the D-pathway mechanism (Belevich et al., 2010).

Correlated internal electron and proton transfer reactions for the O→E transition, tracked in real time by absorption spectroscopic and electrometric techniques, identified the reaction step during which pumping protons are transferred from the N-side to the proton-loading site. The reaction step was coupled with the reduction of heme *a*. However, the electrometric technique is not able to assign the location of the pumping-proton transfer pathway (Belevich, et al., 2007).

##### 4.2.2.5 The decoupling mechanism

Several decoupling mutations in the D-pathway have been reported as experimental results supporting the proposal that D-pathway transfers both pumping and water-forming protons as described in the last paragraph of Section 4.2.1.2. However, it is not clear how the mutated residue stop selectively the pumping protons. Simulation analyses have been conducted, assuming that these decoupled mutations lower the energy barrier that prevents back-leakage of pumping-protons at the pumping proton-loading site from which protons are actively released to the P-side (Liang et al., 2016; 2017; Siegbahn and Blomberg, 2014). The barrier preventing pumping-proton back-leakage is indispensable for any proton-pumping system, since the proton back-leakage process is highly exergonic. They found that a proton transfer barrier existed in the region including several decoupling mutation sites in the wild type CcO and that the barrier was lowered by these mutations, making the rate of pumping-proton back-leak significantly faster than that of the pumping-proton transfer to the proton loading site (Liang et al., 2017; Siegbahn and Blomberg, 2014). Thus, they proposed that the proton pump is abolished by the increase in the proton back-leak rate (Liang et al., 2017). This proposal also indicates that pumping-protons are transferred through the D-pathway.

It has been shown that K319M (in bovine numbering) mutant strongly slows down the A→Pr transition so that Pr is undetectable, resulting in a direct A→F transition. This result suggests that the electron transfer from heme *a* to heme *a*_3_ upon formation of Pr from A must be charge compensated by K319 movement. The necessity of the charge compensation for the A→Pr transition was confirmed by the introduction of a decoupling mutation to the K319M mutant. In fact, K316M/N98D and K316M/N98T mutants restored the Pr→F transition (Vilhjálmsdóttir et al., 2020). These results suggest that the rapid introduction of positive charge (pumping proton) to the proton-loading site near the Fe*a*_3_ provides the charge compensation for the rapid Pr formation, indicating that pumping protons can reach the proton-loading site through the D-pathway. In other words, this finding seems to support the D-pathway mechanism. However, an alternative interpretation, based on the H-pathway mechanism, would be as follows: As described in Section 4.2.2.3. The function of E242, these decoupling mutations, N98D and N98T, decouple the proton-pump function from electron transfer by inducing a translational shift of the heme *a*_3_ plane. Thus, these mutations, in addition to K316M mutation, restore the electron transfer rate from heme *a*_3_ to heme *a* for Pr-formation by the decoupling so that the Pr intermediate is detectable in these double mutants. Thus, these double mutation analyses do not disprove the H-pathway mechanism.

##### 4.2.2.6 Attempts to identify the structure and location of the proton-loading site

In the D-pathway mechanism, the pumping proton at the loading site is actively transferred to the P-side via electrostatic repulsion from the water-forming proton that arrives at the O_2_-reduction site. Therefore, the proton-loading site must be sufficiently close to the O_2_-reduction site to experience this electrostatic interaction. At the same time, an effective barrier that prevents spontaneous leakage of pumping protons from the loading site to the O_2_-reduction site, which is highly exergonic, is essential for efficient energy transduction in the CcO reaction (Shimada et al., 2023a; Williams, 1995). For successful proton pumping, the pumping proton must reach the loading site before the arrival of the water-forming proton. To prevent its unintended transfer to the O_2_-reduction site, it has been proposed that the pumping proton transfer occurs much faster than the water-forming proton transfer, a mechanism termed “kinetic gating” (Popović and Stuchebrukhov, 2004; Popović and Stuchebrukhov, 2012). However, even under this model, the pumping proton must still await the arrival of the water-forming proton to receive electrostatic repulsion for proton pumping to the P-side. Notably, the proposed kinetic-gating mechanism does not include any strategy to prevent leakage of the pumping proton from the loading site to the O_2_-reduction site. Despite extensive efforts, the pumping-proton loading site in the D-pathway has not been experimentally identified, primarily due to strong mutual coupling among candidate sites. Simulation analyses have suggested three candidates: the two propionate groups of heme *a*_3_ and H291, which coordinates Cu_B_ (Popović, 2013; Sugitani et al., 2008). However, all of these candidates are located too close to the O_2_ reduction site (Fe_*a*3_ and Cu_B_) to provide any structural feature to serve as effective barriers that prevent the flow of pumping protons at the candidate sites into the O_2_-reduction site. Moreover, current X-ray structures do not reveal any such structural feature near the O_2_ reduction site. This issue of preventing pumping-proton leakage from the pumping-proton loading site to the O_2_ reduction site in the D-pathway has not been adequately addressed in discussions of the D-pathway mechanism (Saura et al., 2022; Wikström et al., 2018).

##### 4.2.2.7 Pumping-proton exit site and system for preventing pumping-proton back leak for D-pathway mechanism

In addition to the proton-loading site in the D-pathway mechanism, the proton exit site remains experimentally unidentified. Some simulation trials aimed at searching the exit site for protons and water to the P-side surface have proposed multiple potential exit pathways to the P-side surface (Cai et al., 2018; Popović and Stuchebrukhov, 2005). The exit site must include a mechanism to prevent proton back-leakage from the P-side, which is highly exergonic. As no redox-coupled conformational changes have been observed at these proposed sites for proton exit, hydration state changes have been suggested as a potential gating mechanism (Cai et al., 2018). X-ray structural results of CcOs reported thus far show no possible candidate for the prevention of back-leak of protons from the D-pathway in contrast to the case of the H-pathway.

##### 4.2.2.8 The conservativity in H-pathway structure

As mentioned in Results, CcOs in B and C families, in which mutations in K-pathway block both oxidative and reductive phases, while any mutations in the putative D-pathways do not show any significant inhibitory effect (Chang et al., 2009; Han et al., 2011). Thus, all the water-forming protons are transferred through the K-pathway. However, they retain proton pumping function, though the coupling efficiency is lower than that of the A-family CcOs (H^+^/e^−^ = 0.5 vs. 1.0) (Chang et al., 2009; Han et al., 2011). As mentioned in Results, the X-ray structure of the H-pathway structure is conserved in all three CcO families, though some evolutionary variation is detectable, while the active D-pathway structure is conserved only in the A-family. Furthermore, no possible proton pumping system other than H-pathway is detectable in the X-ray structures of B- and C-family CcOs, reported thus far (Chang et al., 2009; Han et al., 2011). It is noteworthy that a resonance Raman study (Egawa et al., 2013) and X-ray structural analyses on bovine heart CcO under various oxidation and ligand-binding states (Shimada et al., 2018; 2020; 2021; 2023b) also support the H-pathway mechanism.

## 5 Conclusion and future works

Assuming that the 3D structures of CcO that are critically involved in the basic function, proton pumping coupled with O_2_ reduction, are evolutionarily conserved, we searched for evolutionary conservation in the 3D structures likely to be involved in proton pumping following the H-pathway mechanism, and found that the basic structures indispensable for proton pumping through the H- pathway are conserved in all three CcO families. This strongly suggests that the H-pathway pumps protons. For comparison, we also examined experimental results obtained from X-ray structural and mutational analyses, from which the D-pathway mechanism has been proposed, and we showed that these experimental results are insufficient to exclude the possibility that the H-pathway pumps protons. In 1995, R. J. P. Williams stated the following: “The problem path is the one that involves pumping of protons across the membrane (one way only). These proton movements must be tightly coupled to redox changes and gated. They must not reach the dioxygen reduction site because this would uncouple their obligatory separate path.” (Williams, 1995). The D-pathway mechanism, which proposes that both proton pathways for pumping and water formation are included in a single pathway, does not fulfill Williams’s requirement for a proton-pumping system, whereas the pumping-proton transfer pathway through the H-pathway is structurally well separated from the transfer pathway for water-forming protons. In this article, we have deduced the above conclusions solely from 3D structural data deposited in the PDB, without applying any simulation analyses. Thus, examination of these experimentally derived conclusions by simulation analyses would provide various proposals for novel experimental approaches to improve our understanding of the proton-pumping mechanism of CcO.

Besides the structural conservation in the basic function of CcO, as described above, various structural diversities in CcOs provide many critical insights into the reaction mechanism of CcO. For example, heme *a* in the A-family CcOs is replaced with heme *b* in the B- and C-family CcOs, while the C-family CcOs have heme *b*_3_ instead of heme *a*_3_. However, all three types of CcO pump protons coupled with O_2_ reduction, although the energy coupling efficiency is not completely conserved. Thus, the existence of the formyl and hydroxy farnesylethyl groups in the A-family CcOs is still a big enigma. Furthermore mammalian CcO has 10 nuclear-coded subunits and three mitochondrial gene-coded subunits. The X-ray structures of the 10 nuclear-coded subunits themselves suggest no possible functional role in the enzyme reaction. However, serious human clinical effects induced by genetic mutations in these subunits have shown the critical involvements of these subunits in the enzyme function by controlling subunit assembly processes (Brischigliaro and Zeviani, 2021), even before the X-ray structures of these subunits were determined. (Tsukihara, et al., 1996). Recent research progress on the CcO-deficient diseases is remarkable (Brischigliaro and Zeviani, 2021; Thind et al., 2024). In fact, if these clinical findings were not obtained, the existence of these subunits would still have remained a complete enigma.

## Data Availability

The original contributions presented in the study are included in the article/Supplementary Material, further inquiries can be directed to the corresponding author.
