# Supplementary material for: The three-dimensional structure of a proton-pumping pathway, the H-pathway, is evolutionarily conserved in all three families of cytochrome c oxidase
Source: Front Chem. 2026 Jan 30;13:1645343. doi: 10.3389/fchem.2025.1645343 (PMC12901375; doi:10.3389/fchem.2025.1645343)
Supplement: Supplementary file 1 [file Supplementaryfile1.docx]

**Supplementary material**

**Supplementary text 1. A time- resolved ATR-FTIR analysis for the identification of the residues involved in the proton-transfer process by using D-pathway mutant CcOs.**.

In the D91N mutant of *P. denitrificans*, the F→O transition is slow enough to follow with an FTIR system with about 46-msec time resolution (slow phase) as reported (Gorbikova, et al., 2007). The F→O transition showed a positive peak at 1743 cm^-1^, which is assignable to essentially full protonation of E242 (in bovine number). The difference spectrum between the CO-inhibited fully reduced minus F forms, equivalent to the R→F difference spectrum, was obtained by subtracting the initial spectrum obtained after flash-photolysis from the spectrum before flash-photolysis (fast phase). The spectrum showed a trough at 1735 cm^-1^, due to complete deprotonation of E242 during the transition from R to F. This finding indicates that protons for R → F transition are donated from E242 (Gorbikova, et al., 2007). Under the D-pathway mechanism, this full deprotonation of E242 is assigned to pumping-proton transfer from E242 to a proton loading site. By using D91N and D91N/Y19F mutants, kinetic P-F and F-O difference FTIR spectra were obtained, showing IR absorbance changes induced upon Pr to F and F to O transitions, respectively. A peak at 1278 cm^-1^ appears upon F-formation from Pr and disappears upon F-O transition. The 1278 cm^-1^ peak is assignable to approximately 50% of the deprotonated tyrosine (tyrosine-O^-^). Thus, the FTIR results indicate that about 50% of a tyrosine residue is deprotonated upon formation of the F-form from the P-form, while the deprotonated tyrosine is protonated upon formation of the O-form from the F-form. Belevich et al assigned these spectral changes to Y19 and proposed that they confirm the role of Y19 as the water forming proton donor for the Pr → F transition, as proposed by electrometric analysis, (Belevich et al., 2010).

However, as described in the Introduction, Y244 is the water-forming proton acceptor in the Pr → F transition. Thus, when Y19-OH transfers a water-forming proton to Y244 in Pr, no band intensity change in the 1278 cm^-1^ band region should appear, which is clearly inconsistent with the experimental results showing a 1278 cm^-1^ band increase corresponding to 50% deprotonation of one tyrosine residue. This finding suggests that the1278 cm^-1^ band of Y244O^-^ in the Pr form is broadened significantly, due to tight interaction with heme *a*_3_. An alternative interpretation is that the water-forming proton from Y19 is not received by Y244, but by Cu_B_^2+^-OH^-^, as has been suggested (Wikstrom, et al, 2018), although high resolution X-ray structural studies (Shimada et al, 2020) and magnetic circular dichroism studies(Jose, et al, 2021) do not support this alternative, as described in Introduction. This proposal for the role of Y19 as the proton donor to Y244 is based on the D-pathway mechanism. However, an equally reasonable interpretation of the above IR results is possible under the H-pathway mechanism, as follows: in the H-pathway mechanism, protons in the D-pathway are not used for pumping. Thus the water-forming protons for the Pr → F transition are provided by E242, not by Y19. In the Pr → F transition, approximately 50 % of the E242 protons are transferred to protonate the Y244-O^-^ of Pr to produce 50 %of the F-form, without causing any intensity change in the1278 cm^-1^ band, as in the preceding interpretation based on the D-pathway mechanism. It has been shown that pumping-proton collection from the N-side is coupled with the Pr → F transition (Faxén, et al, 2005), suggesting that deprotonation of E242 is likely to trigger proton collection to the pumping proton pool system as given in Fig. 8. Thus, during the Pr → F transition, fully deprotonated E242 (or the negatively charged E242) induces approximately 50% deprotonation of one of the tyrosine residues in the proton pool system.in order to facilitate proton collection to the proton pool below the proton gate of the water channel, while in the F → O transition the deprotonated tyrosine residues are re-protonated. These deprotonation and re-protonation events produce the intensity change in the1278 cm^-1^ band. Therefore, both D- and H pathway mechanisms are able to interpret the ATR-FTIR results equally reasonably. In other words, the ATR-FTIR results do not conclusively support the D-pathway mechanism.
